# Limiting ER-associated degradation capacity triggers acute and chronic effects on insulin biosynthesis

**DOI:** 10.1172/JCI187341

**Published:** 2025-11-18

**Authors:** Anoop Arunagiri, Leena Haataja, Maroof Alam, Noah F. Gleason, Emma Mastroianni, Chao-Yin Cheng, Sami Bazzi, Jeffrey Knupp, Ibrahim Metawea, Anis Hassan, Dennis Larkin, Deyu Fang, Billy Tsai, Ling Qi, Peter Arvan

**Affiliations:** 1 Division of Metabolism, Endocrinology & Diabetes, University of Michigan Medical Center, Ann Arbor, Michigan, USA.; 2 Department of Cell and Developmental Biology, University of Michigan, Ann Arbor, Michigan, USA.; 3 Department of Pathology, Feinberg School of Medicine, Northwestern University, Chicago, Illinois, USA.; 4 Department of Molecular Physiology and Biological Physics, University of Virginia Medical School, Charlottesville, Virginia, USA.

**Keywords:** Cell biology, Endocrinology, Beta cells, Insulin

## Abstract

In pancreatic β cells, misfolded proinsulin is a substrate for ER-associated protein degradation (ERAD) via HRD1/SEL1L. Alternately, β cell HRD1 activity is reported to improve, or impair, insulin biogenesis. Further, while β cell SEL1L deficiency causes HRD1 hypofunction and diminishes islet insulin content, reports conflict as to whether β cell ERAD deficiency increases or decreases proinsulin levels. Here, we examined β cell–specific *Hrd1*-KO mice (chronic deficiency) and rodent (and human islet) β cells treated acutely with HRD1 inhibitor. *β-Hrd1*–KO mice developed diabetes with decreased islet proinsulin, yet a relative increase of misfolded proinsulin redistributed to the ER. They also showed upregulated biochemical markers of β cell ER stress and autophagy, electron microscopy evidence of ER enlargement and decreased insulin granule content, and increased glucagon-positive islet cells. Misfolded proinsulin was also increased in islets treated with inhibitors of lysosomal degradation. Preceding any loss of total proinsulin, acute HRD1 inhibition triggered increased nonnative proinsulin, increased phospho-eIF2α with inhibited proinsulin synthesis, and increased LC3b-II (the abundance of which requires expression of ΣR1). We posit a subset of proinsulin molecules undergo HRD1-mediated disposal. When HRD1 is unavailable, misfolded proinsulin accumulates, accompanied by increased phospho-eIF2α that limits further proinsulin synthesis, plus ΣR1-dependent autophagy activation, ultimately lowering steady-state β cell proinsulin (and insulin) levels and triggering diabetes.

## Abstract 2

We limited ERAD capacity in β-cell-specific HRD1-KO mice, or HRD1 inhibitor-treated β-cells; predisposing to ER stress, diminished proinsulin synthesis, and SigmaR1-dependent activation of autophagy.

## Graphical abstract

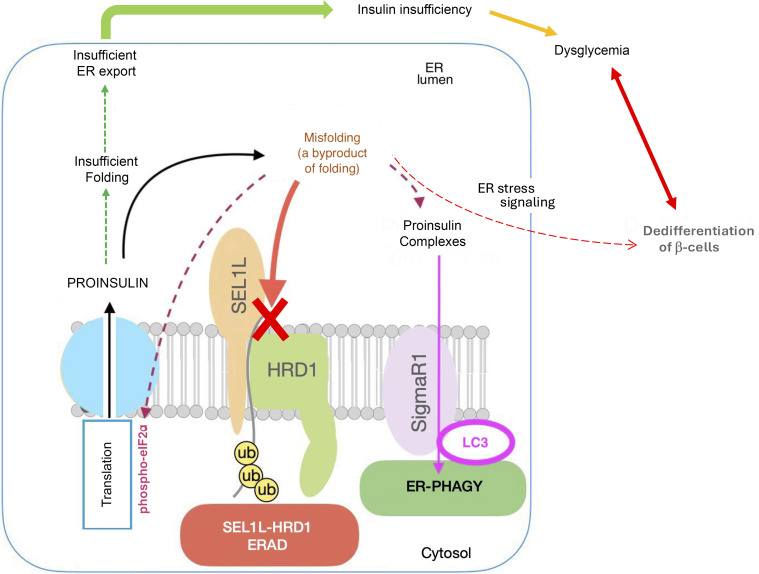

## Introduction

Misfolded mutant or WT proinsulin has long been posited to be a substrate of ER-associated degradation (ERAD) (1–4) via the SEL1L/HRD1/p97 pathway (5–7) leading to proteasomal degradation (8, 9). In this pathway, HRD1 participates in retrotranslocation of ERAD substrates from the ER lumen, coupled with substrate ubiquitylation (10); SEL1L participates as a HRD1 partner protein and cofactor (11); and p97 participates in extraction of ubiquitylated substrates from the ER membrane for delivery to proteasomes in the cytosol (12).

In the last 6 years, studies have begun to probe the importance of this pathway for proper pancreatic β cell function. Despite considerable effort, confusion has remained over the directionality of the impact of increased or decreased ERAD function in pancreatic β cells and its implications for the development of diabetes. One group has reported that increased β cell expression of HRD1 (such as may occur in type 2 diabetes or in mouse models of the disease) triggers impaired insulin secretion, whereas impaired ERAD capacity by HRD1 knockdown (KD) improves blood glucose control (13). However, a state of HRD1 KD exists in all cells upon diminished expression of SEL1L — its interaction confers HRD1 stability in mammalian cells (11, 14, 15) and in yeast (16) — but rather than improving glucose-stimulated insulin secretion (GSIS) and blood glucose homeostasis, deficient ERAD capacity was found to result in diminished GSIS with development of whole animal glucose intolerance (6). Indeed, mice genetically deficient for β cell expression of *Sel1L* have been reported to have normal embryonic development of islets but proceed to severe diabetes with evidence of altered islet cell transcriptional states (17), consistent with islet cell heterogeneity that has been described in several models of full-blown diabetes in rodents (and in humans) (18). Yet, there remains considerable discrepancy even between the 2 groups that have been studying deficient ERAD capacity caused by β cell *Sel1L* deletion. Both groups identified insulin deficiency, but one found that it was accompanied by a large steady-state increase of β cell proinsulin (6), while the other found a marked decrease in proinsulin content (19). Both studies were performed in the same mouse genetic background, with the only difference being the use of a transgenic *RIP-Cre* in the first ERAD-deficient model (6) rather than a knockin *Ins1*-Cre in the other (17, 19).

Here, we sought to clarify the impact of ERAD capacity and report to our knowledge the first β cell–specific deletion of *Hrd1* (also known as *Synv1*), along with appropriate controls, demonstrating that islet proinsulin levels (as well as insulin levels) are unequivocally low in *β-Hrd1-*KO mice. (We’ve intentionally used the transgenic *RIP-Cre*; thus, this cannot be the explanation for prior discrepancies.) We find that, beyond the islet cell heterogeneity appearing in the islets of *β-Hrd1-*KO mice that consistently develop hypoinsulinemic diabetes, there is a notable increase in the phosphorylation state of eIF2α, which is known to limit proinsulin biosynthesis, as well as an activation of autophagy that participates in proinsulin turnover. These effects occurring upon chronic adaptation to β cell–specific loss of HRD1-mediated ERAD capacity are also recapitulated within as little as 2 h of acute chemical HRD1 inhibition, even before any loss of total proinsulin is detected, and the extent of autophagy activation is dependent upon the expression of the ER membrane protein encoded by *SigmaR1* (ΣR1), which has recently been identified as a required element for biosynthesis of the critical autophagy factor LC3 (20). Thus, in addition to effects on the differentiation state of pancreatic β cells, ERAD capacity links clearance of misfolded proinsulin to biosynthesis of new proinsulin and the extent of autophagy activation, ultimately regulating insulin content and the risk of developing insulin deficiency and diabetes.

## Results

### β Cell proinsulin content is diminished in β-Hrd1-KO islets.

**Figure 1:**
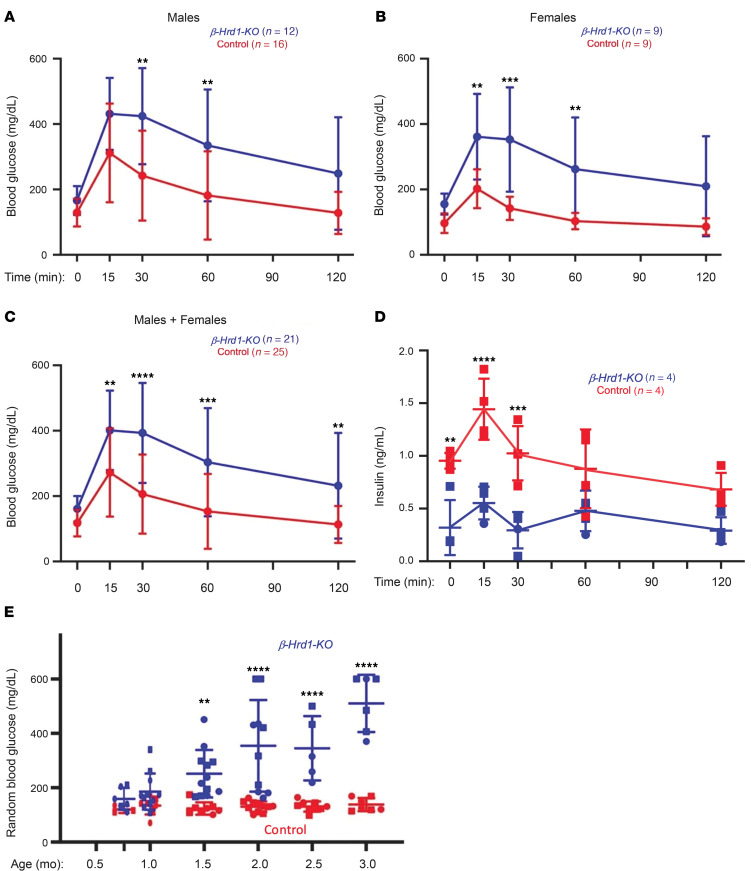
Intraperitoneal glucose tolerance test in 6- to 8-week-old *β-Hrd1*–KO or control mice on normal chow. (**A**) Males (mean ± SD). (**B**) Females (mean ± SD). (**C**) Combined males + females from **A** and **B**. (**D**) GSIS (control group, 4 males; *β-Hrd1*–KO, 3 males + 1 female; mean ± SD). (**E**) Random blood glucose measurements as a function of age in randomly selected males plus females (up to 10 per group). Data were analyzed by 2-way ANOVA with Šidák’s multiple-comparison test; **P < 0.01, ***P < 0.001, ****P < 0.0001.

We generated mice with β cell–specific deletion of *Hrd1* (official gene name: *Synv1*) using a *RIP-Cre* transgene (21). To exclude off-target effects (22), we used *RIP-Cre*–positive *Hrd1* heterozygotes as controls. As islets are not purely β cells, the result of immunoblotting *β-Hrd1*–KO islets is compatible with complete loss of β cell HRD1 expression accompanied by a notable decrease of islet insulin (a representative blot is shown in Supplemental Figure 1A; quantitation of insulin content is shown by ELISA in Supplemental Figure 1B; supplemental material available online with this article; https://doi.org/10.1172/JCI187341DS1) as well as an increase of IRE1α and SEL1L (Supplemental Figure 1C), as reported in other HRD1-deficient cell types (23). *β-Hrd1*–KO mice did not show a decrease of islet size relative to controls (Supplemental Figure 2) but developed glucose intolerance in males (Figure 1A), females (Figure 1B), and both combined (Figure 1C). Impaired glucose tolerance was coupled with inadequate insulin secretion in vivo (Figure 1D), and when considered as a fraction of (the low) islet insulin content (Supplemental Figure 1B), glucose-augmented insulin secretion (Supplemental Figure 1D) was not decreased from isolated islets of *β-Hrd1*–KO mice. Nevertheless, within the first 6 postnatal weeks, the body weight of *β-Hrd1*–KO mice began to grow at a diminished rate compared with controls (Supplemental Figure 3A) and the animals developed random hyperglycemia (Figure 1E). Nevertheless, serum insulin did not rise in response to the increased blood glucose (in fact, it declined), such that the serum insulin/glucose ratio dropped without ever going through a transient hyperinsulinemic period (Supplemental Figure 3B).

**Figure 2:**
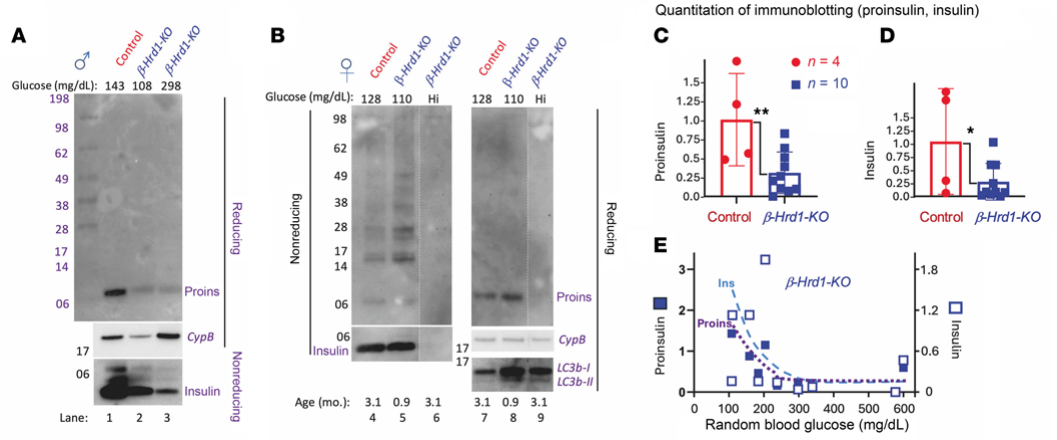
Proinsulin and insulin deficiency develops in *β-Hrd1*–KO mice. (**A**) Immunoblotting after SDS-PAGE of islet lysates from control or *β-Hrd1*–KO male mice (lanes 1–3, random blood glucose values are indicated at top). Reducing gel (above) highlights total proinsulin level (CypB is a loading control); nonreducing gel (below) highlights total mature (2-chain) insulin. (**B**) Immunoblotting after SDS-PAGE of islet lysates from control or *β-Hrd1*–KO female mice (lanes 4–6 or 7–9; random blood glucose values indicated at top). Reducing gel (above) highlights total proinsulin level (CypB is a loading control); LC3b is shown below in lanes 7–9. Nonreducing gel highlights nonnative proinsulin molecules in disulfide-linked complexes (above in lanes 4–6); nonreducing gel (below) highlights total mature insulin. (**C**) Quantitation of immunoblots indicating relative proinsulin content (normalized to HSP90) in islets of *β-Hrd1*–KO mice (each point represents a different animal; squares = males, circles = females; *n* is indicated). (**D**) Quantitation of immunoblots indicating relative content of mature insulin (normalized to HSP90) in islets of *β-Hrd1*–KO mice (each point represents a different animal; squares = males, circles = females; total *n* is indicated). Data in **C** and **D** were analyzed by unpaired 2-tailed *t* test; *P < 0.05, **P < 0.01. (**E**) The data in **D** and **E** were replotted relative to the random blood glucose of each animal at the time of euthanasia (proinsulin = closed squares; insulin = open squares).

Although proinsulin is an ERAD substrate, we did not observe the increase in islet proinsulin content previously reported by one group using *RIP-Cre* to generate ERAD deficiency in *β-Sel1L*–KO mice (6). Using heterozygous littermate controls, side-by-side comparisons demonstrated proinsulin (and insulin) deficiency (Figure 2A, lane 3, quantitation in Figure 2, C and D), which worsened upon development of diabetes in *β-Hrd1*–KO mice (Figure 2E), although rarely (using a preweaning *β-Hrd1*–KO animal) islet proinsulin (and insulin) levels appeared similar to those of control animals (e.g., Figure 2B, lanes 5 and 8). In the islets of *β-Hrd1*–KO mice with full-blown diabetes, both islet proinsulin and insulin were always low (Figure 2B, lanes 6 and 9). Additionally, islets of *β-Hrd1*–KO mice stored less IAPP, as detected by quantitative Western blotting (Supplemental Figure 3C), indicating that the phenotype attributable to a deficiency of β cell ERAD capacity is not selective for insulin alone.

### Impact of β-Hrd1–KO on islet morphology.

**Figure 3:**
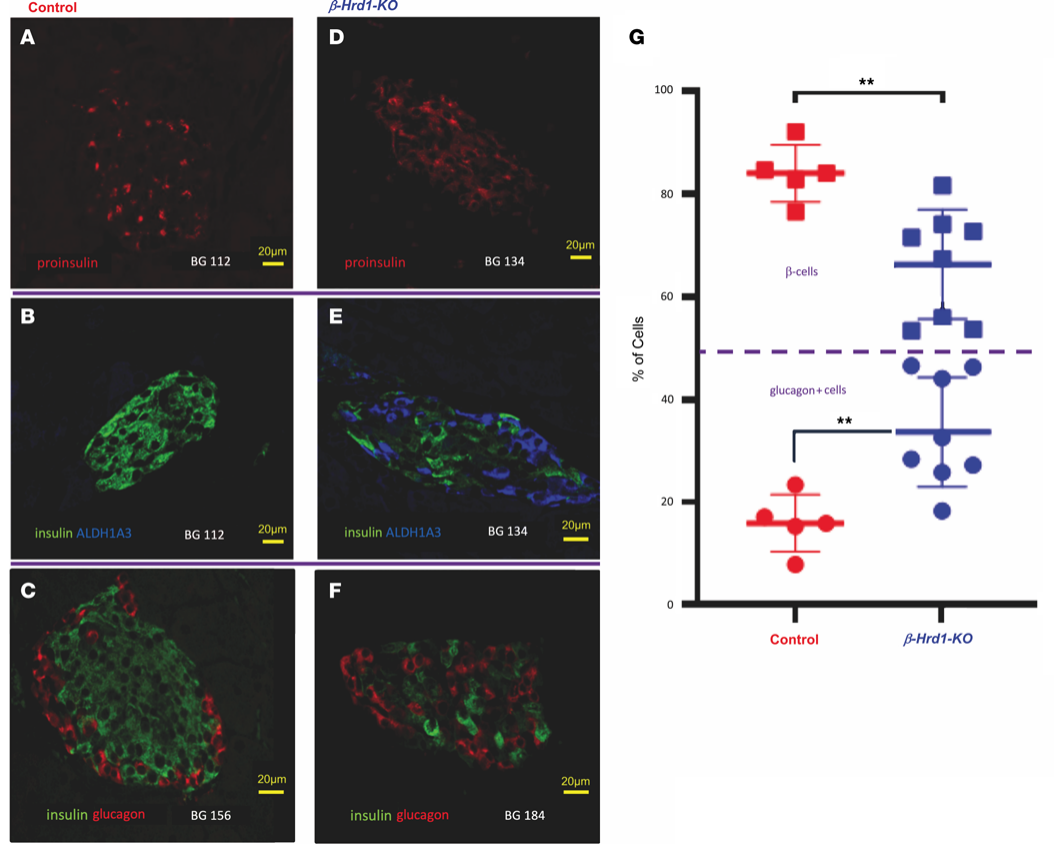
Proinsulin, insulin, ALDH1A3, and glucagon in pancreatic tissue sections from *β-Hrd1*–KO mice. (**A** and **D**) Proinsulin immunofluorescence from control and *β-Hrd1*–KO, respectively (control group, *n* = 9; *β-Hrd1*–KO, *n* = 7). (**B** and **E**) ALDH1A3 and insulin from control and *β-Hrd1*–KO, respectively (control group, *n* = 4; *β-Hrd1*–KO, *n* = 7). (**C** and **F**) Glucagon and insulin immunofluorescence from control and *β-Hrd1*–KO, respectively. Random blood glucose (BG) values are indicated at the bottom of each image. Scale bars: 20 μm. (**G**) The fraction of glucagon-positive cells versus cells positive for either proinsulin or insulin (sum of both, with each β cell counted only once) from control (random blood glucose mean = 97 mg/dL) and *β-Hrd1*–KO (random blood glucose mean = 257 mg/dL). For quantitation, the 2 types of islet cells together are referred to as 100% (*n* = 4 animals per group, each point represents islets in 1 section; unpaired 2-tailed *t* test, **P < 0.01).

In control islets, proinsulin is concentrated in a juxtanuclear pattern (Figure 3A) thought to represent the Golgi region where immature secretory granules are formed (24, 25). However, in *β-Hrd1*–KO islets, proinsulin tended to have a more diffuse cytoplasmic distribution (Figure 3D), similar to that of the ER (Supplemental Figure 4A). Moreover, ALDH1A3 immunofluorescence (Figure 3B) increased in the islets of *β-Hrd1*–KO mice (Figure 3E) — an indicator of pancreatic islet dedifferentiation (17, 26, 27) — although some of these cells still retained expression of proinsulin and insulin protein (Supplemental Figure 4B). Additionally in *β-Hrd1*–KO islets, there was a notable increase in the fraction of glucagon-positive cells (Figure 3, F vs. C). This increase was accompanied by a fractional decrease of insulin-positive cells (Figure 3E, quantified in Figure 3G) and a higher fraction of β cells that exhibited weaker insulin immunostaining, consistent with a decrease of islet insulin (Figure 2D and Supplemental Figure 1B) and an increase in islet cell heterogeneity suggested to be linked to diabetes in humans and several rodent models (18, 28–33).

**Figure 4:**
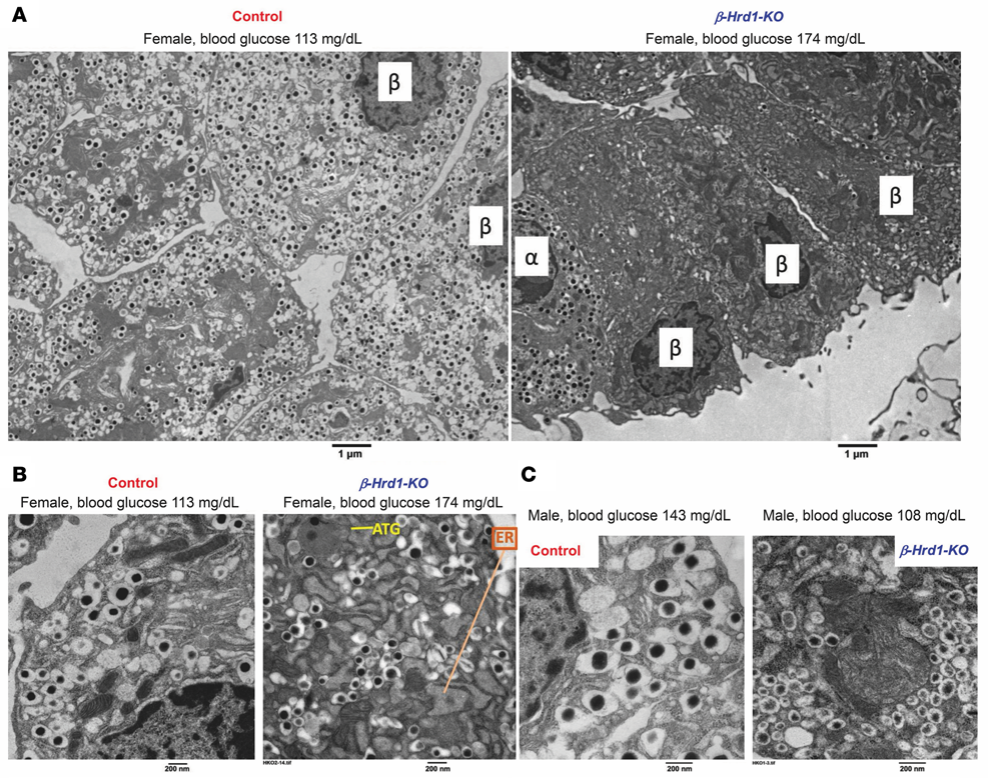
Electron microscopy of *β-Hrd1*–KO islet sections. (**A**) Low-power transmission electron microscopy images of islet tissue sections from control and *β-Hrd1*–KO females (random blood glucose indicated above). The α and β cells are marked accordingly. (**B**) Higher magnification of islet sections from mice in **A**. (**C**) Higher magnification of islet sections from control and *β-Hrd1*–KO males (random blood glucose indicated above). Images highlight differences in secretory granule size between control and *β-Hrd1*–KO animals; ER and autophagosome (ATG) are indicated in **B** (*n* = 3 animals per group; quantitation of secretory granule size is shown in Supplemental Figure 5). Scale bars: 1 μm (**A**), 200 nm (**B** and **C**).

The basis for diminished insulin immunostaining was made clearer upon examination by transmission electron microscopy. At low magnification, insulin granules in the β cells of *β-Hrd1*–KO islets were hard to discern (Figure 4A). At a higher magnification, it was clear that in both females (Figure 4B) and males (Figure 4C), the diameters of individual insulin secretory granules in β cells from *β-Hrd1*–KO mice are approximately 55% smaller in size than their control counterparts (Supplemental Figure 5; corresponding to a reduction of cross-sectional area of individual granules of ~80% and a reduction of spherical volume of ~90%). Such microgranules have been reported in other diabetes models, where they are associated with diminished β cell insulin content (34, 35). Moreover, autophagosomes were detected in the cytoplasm (ATG, Figure 4B) and the ER was distended (as highlighted in Figure 4B), suggesting ER stress.

### ER response in β-Hrd1–KO islets.

**Figure 5:**
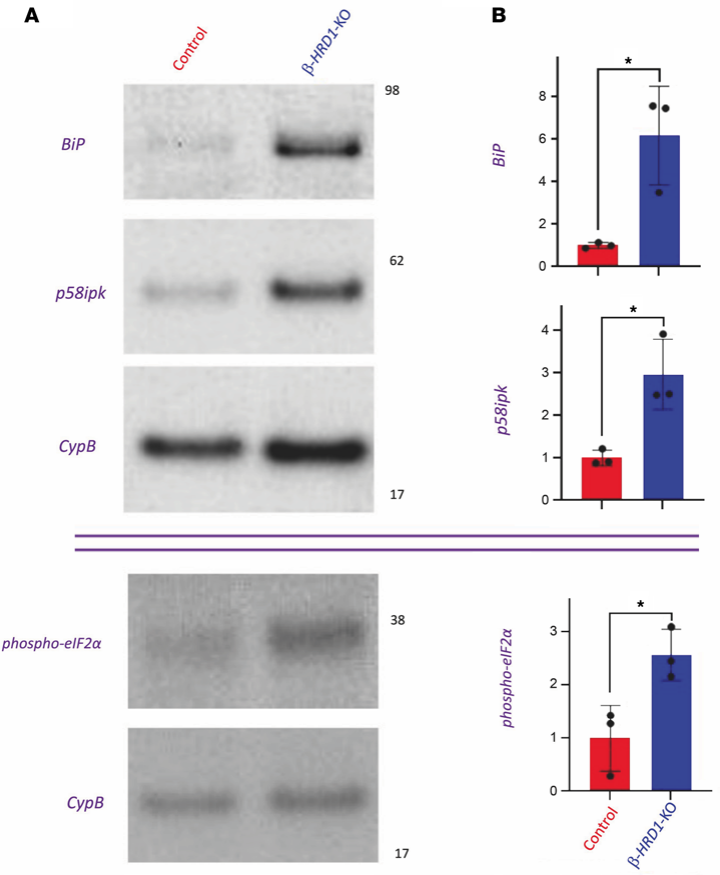
Steady-state levels of BiP, p58^ipk^ (ERdj6), and phospho-eIF2α in islets of *β-Hrd1*–KO mice. (**A**) Immunoblotting of BiP, p58^ipk^ (ERdj6), and phospho-eIF2α antigens compared with CypB as a loading control. (**B**) Quantitation of BiP and p58^ipk^ (ERdj6) normalized to CypB (*n* = 3 per group; mean ± SD; unpaired 2-tailed *t* test; *P < 0.05); mean random blood glucose for control cohort = 174 mg/dL and for *β-Hrd1*–KO cohort = 263 mg/dL. Quantitation of phospho-eIF2α normalized to CypB (*n* = 3 per group; mean ± SD; unpaired 2-tailed *t* test; *P < 0.05); mean random blood glucose for control cohort = 147 mg/dL and for *β-Hrd1*–KO cohort = 119 mg/dL.

Even before the development of diabetes, islets of *β-Hrd1*–KO mice exhibited significant elevation of the ER luminal chaperone, BiP, and cochaperone, p58^ipk^ (Figure 5A, quantified in Figure 5B). Moreover, *β-Hrd1*–KO islets exhibited an increase of phospho-eIF2α (Figure 5A, quantified in Figure 5B), which is known to limit proinsulin synthesis (36–39). To check this, we performed metabolic pulse-chase studies with ^35^S–amino acids and observed a decrease in proinsulin biosynthesis (as well as subsequent generation of mature insulin) in *β-Hrd1*–KO islets (Supplemental Figure 6A). However, when normalized to TCA-precipitable cpm, the magnitude of the decrease in proinsulin biosynthesis amounted to only 15%, whereas the steady-state proinsulin level decreased to a much greater degree (Supplemental Figure 6B), suggesting that either pulse-labeled proinsulin normalized to TCA-precipitable cpm underestimates the full magnitude of the proinsulin synthesis defect or that decreased synthesis is only part of the explanation for the decline of proinsulin levels (both points are investigated further below). Indeed, we noted that activation (lipidation) of LC3b to form LC3b-II, suggesting activation of autophagic turnover, was also detectable in young nondiabetic *β-Hrd1*–KO islets (Figure 2B, lanes 8 and 9).

### Acute HRD1 inhibition in pancreatic β cells.

**Figure 6:**
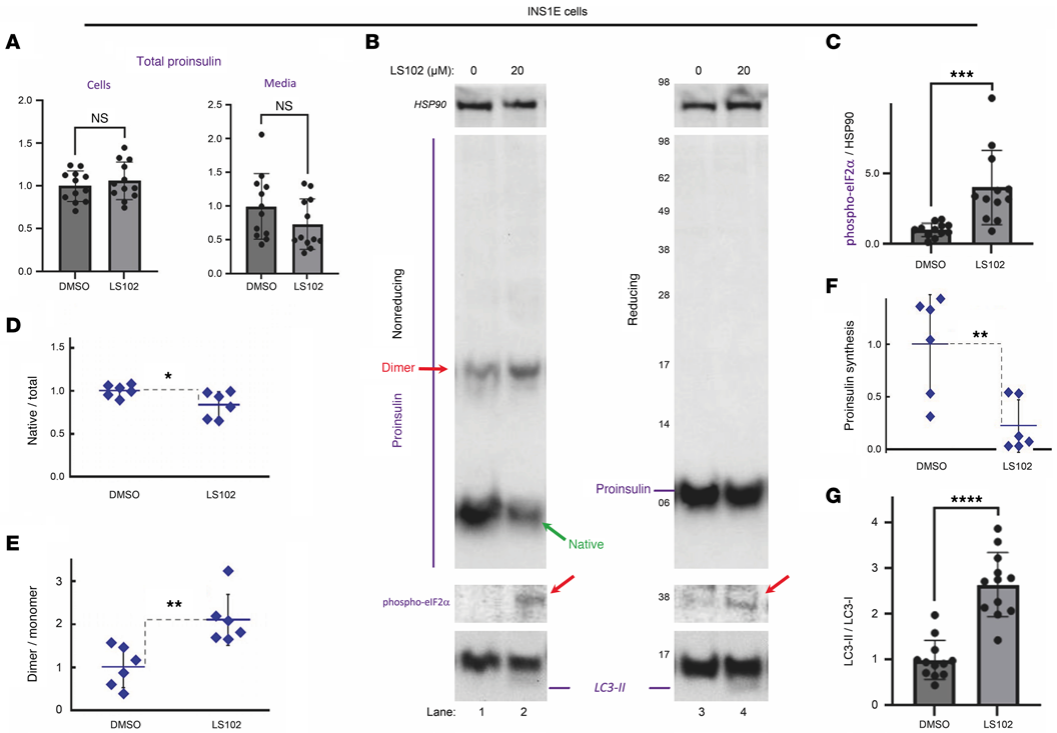
Acute treatment of INS1E (β cell line) with LS102. (**A**) Intracellular (cells, left) and extracellular (media, right) proinsulin from INS1E cells treated with LS102 (20 μM) for 2 h. (**B**) Nonreducing SDS-PAGE and immunoblotting of proinsulin from lysates of LS102-treated INS1E cells (lanes 1 and 2, above) or phospho-eIF2α (middle) and LC3b (bottom). (**C**) Quantitation of phospho-eIF2α (*n* = 12). (**D**) Quantitation of native proinsulin as a fraction of total proinsulin (*n* = 6). (**E**) Quantitation of nonnative proinsulin dimer (as in **B**) relative to approximately 6 kDa proinsulin monomer (*n* = 6). (**F**) Quantitation of (pre)proinsulin synthesis, as measured in Supplemental Figure 8 (*n* = 6). (**G**) Quantitation of LC3-II/LC3-I (*n* = 12 independent experiments). All quantitation shown is mean ± SD; unpaired 2-tailed *t* test; *P < 0.05, **P < 0.01, ***P < 0.001, ****P < 0.0001.

The fact that a rare preweaning *β-Hrd1*–KO mouse can exhibit nearly normal proinsulin and insulin levels (Figure 2B, lanes 5 and 8) suggested that diminished proinsulin observed in these chronically ERAD-deficient mice represents an adaptation that develops postnatally. To examine acute effects of HRD1 inhibition, we treated INS1E β cells with LS102 for 2 h. LS102 inhibits Hrd1 autoubiquitination (40) and at 10–30 μM also inhibits the ubiquitination of ERAD substrates (41). LS102 was shown in cultured (human alveolar epithelial) cells to trigger significant inhibition of protein (collagen) secretion (42). Acute ERAD inhibition does not significantly change the content of proinsulin in either β cells or their culture medium (Figure 6, A and B). However, based on a recently improved immunoblotting methodology for detection of native and nonnative proinsulin monomers as well as disulfide-linked dimers (43), it was apparent that upon acute HRD1 inhibition, the population of intracellular proinsulin molecules shifts away from native monomers (Figure 6B, lane 2, quantified in Figure 6D), and an increase in phospho-eIF2α (Figure 6B, lane 4, quantified in Figure 6C) accompanies the increase of misfolded proinsulin (Figure 6E). Unlike previous immunoblotting methodology of nonreduced proinsulin that is supersensitive to intermolecular disulfide-linked complexes (Supplemental Figure 7A), the current methodology (43) more accurately detects monomeric proinsulin species (Supplemental Figure 7B). However, regardless of the immunoblotting method, acute HRD1 inhibition results in accumulation of misfolded proinsulin, and similar behavior (diminished native proinsulin) was observed upon LS102 treatment of (nondiabetic) human islets, accompanied by increased phospho-eIF2α and LC3-II (Supplemental Figure 7C and Supplemental Figure 8). Moreover, after an initial 2 h of protein synthesis inhibition in INS1E cells, a cycloheximide washout protocol detected new proinsulin synthesis as an increase of proinsulin abundance (Supplemental Figure 9, lane 7 vs. 6). However, when accompanied by acute HRD1 inhibition, there was little increase in total proinsulin abundance (Supplemental Figure 9, lane 8 vs. 6), but there was increased proinsulin misfolding (Supplemental Figure 9, lane 4 vs. 2; misfolded proinsulin species highlighted with red arrows) and increased phospho-eIF2α (Supplemental Figure 9, lane 8).

To test directly whether the increased phospho-eIF2α triggered by acute inhibition of HRD1 activity is linked to a decrease of new proinsulin synthesis, we used 2 independent methods. First, we exploited the recently reported simple method in which a brief combined inhibition of the SEC61 translocon (blocking ER translocation) with simultaneous proteasome inhibition allows detection of new synthesis in the form of (pre)proinsulin (38, 44). Using this method, new (pre)proinsulin synthesis was readily apparent in control INS1E cells (Supplemental Figure 10A, lanes 3 and 5), but upon HRD1 inhibition with LS102 (which triggers an increase of phospho-eIF2α; Figure 6C), (pre)proinsulin synthesis was decreased by approximately 60% (Supplemental Figure 10A, lanes 4 and 6, quantified in Figure 6F). Second, using a conventional 15 min pulse-labeling with ^35^S–amino acids, upon acute HRD1 inhibition, proinsulin synthesis (normalized to TCA-precipitable cpm) was decreased by approximately 40% (Supplemental Figure 10B). Because the rise in phospho-eIF2α also affects global protein translation (45), proinsulin synthesis normalized against global protein translation (measured by TCA-precipitable cpm) detected a lower fractional inhibition (Supplemental Figure 10B) than observed by normalizing against a stable loading control protein like HSP90 (Figure 6F); nevertheless, both independent methods indicate diminished proinsulin synthesis following acute HRD1 inhibition.

**Figure 7:**
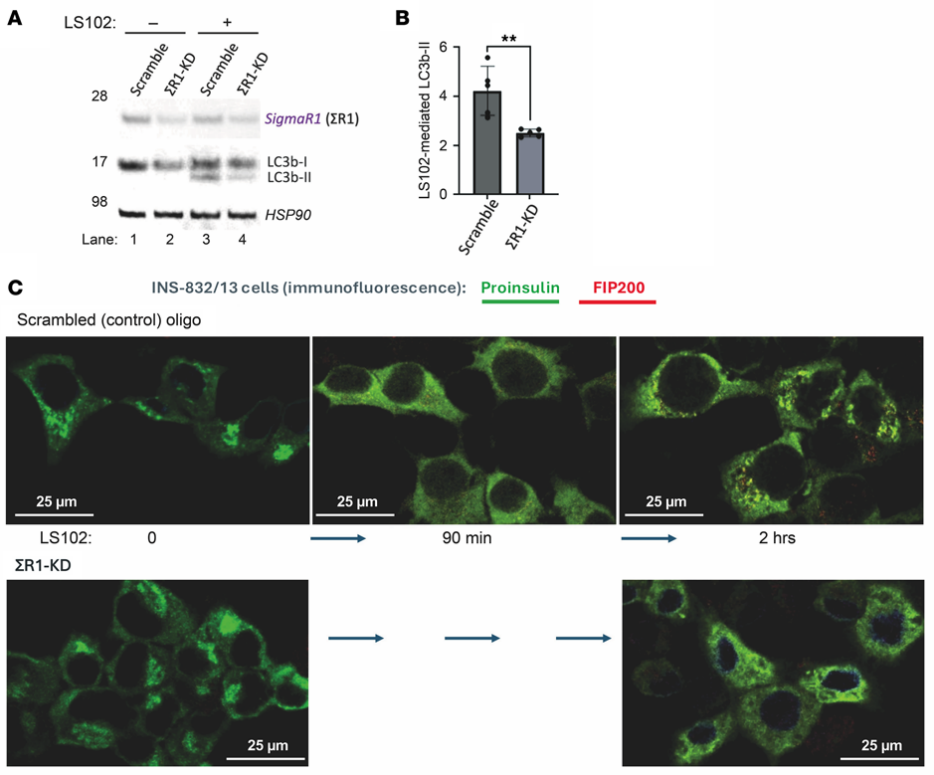
Effects of ΣR1 and LS102 on LC3, and proinsulin distribution in INS1 cells. (**A**) Immunoblotting for ΣR1 or LC3 after LS102 treatment of control INS1E cells (scramble oligo) or ΣR1 KD; Hsp90 was a loading control. (**B**) Quantitation of LC3b-II (normalized to HSP90) in LS102-treated cells after ΣR1 KD (*n* = 5; unpaired 2-tailed *t* test; **P < 0.01). (**C**) INS832-13 cells were transfected with control oligo or ΣR1-KD and after 48 h were treated ± LS102 (20 μM) for up to 2 h (*n* = 4 per group) before fixation, permeabilization, and immunofluorescence for proinsulin (green) and FIP200, a marker of autophagosome formation (red). Scale bars: 25 μm.

Additionally, among the acute responses to diminished HRD1 activity is LC3b activation (lipidation) to LC3b-II (Figure 6G). Recent findings indicate that the ΣR1 gene product promotes translation of LC3b-I at the ER, and this is the substrate for the generation of lipidated LC3b-II (20). Consistent with these observations, upon siRNA-mediated ΣR1 KD in INS1E β cells (Figure 7A), we observed diminished LC3b-I (Figure 7A, lane 2 vs. 1), and upon acute HRD1 inhibition, we observed diminished LC3b-II (Figure 7A, lane 4 vs. 3, quantified in Figure 7B). INS-832/13 β cells, which grow as relatively flat cells that are favorable for immunofluorescence (46), revealed a primary localization of rodent proinsulin in a juxtanuclear pattern, typical of the normal proinsulin distribution in the Golgi region (25), that was not affected by ΣR1 KD (Figure 7C), strongly suggesting that anterograde trafficking of proinsulin to the Golgi complex continues in ΣR1-deficient β cells. Acute HRD1 inhibition with LS102 caused β cells to lose their strong juxtanuclear proinsulin distribution within 90 min (Figure 7C), implying impaired proinsulin anterograde trafficking with diminished concentration in the Golgi complex. By 2 h of LS1012 treatment, a number of small bright puncta newly appeared that were positive both for proinsulin and FIP200 (Figure 7C), a marker of autophagosome formation (47).

The proinsulin-positive puncta that form upon a 2 h treatment with LS102 were found to be calnexin positive, indicating that they derive from the ER compartment (Supplemental Figure 11, lower left panel), and the formation of such ER-derived proinsulin-positive puncta was blocked in ΣR1-deficient cells (Figure 7C and Supplemental Figure 11, lower right panel), with an intracellular distribution of proinsulin that suggests both failure of proinsulin anterograde trafficking and entry into autophagosomes. Together, the data in Figures 7 and Supplemental Figure 11 support that in β cells, LC3b-I levels (and LC3b-II levels after acute HRD1 inhibition) are dependent upon ΣR1 expression, such that when both ERAD and ER-phagy clearance pathways are acutely inhibited (ERAD by LS102 and autophagy by ΣR1-siRNA), proinsulin becomes entrapped in the ER. Thus, acute HRD1 inhibition in β cells is accompanied by an increase of misfolded proinsulin complexes (Figure 6, B and E, and Supplemental Figures 7 and 8) with decreased native proinsulin (Figure 6D), as well as increased phospho-eIF2α (Figure 6C) that is linked to decreased proinsulin biosynthesis (Supplemental Figure 10), accompanied by autophagy activation (Figure 6G). Autophagy captures some proinsulin (Figure 7C) originating in the ER (Supplemental Figure 11), and the extent of capture is dependent upon expression of ΣR1 (Figure 7, A–C, and Supplemental Figure 11). Indeed, in human islets, aberrant disulfide-linked proinsulin complexes appear destined for a lysosomal fate, as these complexes accumulate upon treatment with lysosomal inhibitors (e.g., chloroquine in Supplemental Figure 12A, quantified in Supplemental Figure 12B and associated with increased LC3-II in Supplemental Figure 12C), events that also occur in *β-Hrd1*–KO and even WT murine islets (Supplemental Figure 12, D and E). Thus, when β cell HRD1-mediated ERAD is deficient, in addition to islet cell heterogeneity (Figure 3) and ER stress response with increased phospho-eIF2α (Figure 5) accompanied by decreased proinsulin biosynthesis (Supplemental Figure 6A and Supplemental Figure 10), our data suggest that autophagy is also involved in conveying misfolded proinsulin complexes to their ultimate turnover in lysosomes (Supplemental Figure 12).

## Discussion

SEL1L-HRD1 (plus associated partner proteins) identify and remove many misfolded proteins from the ER (48). Misfolded proinsulin is subject to ERAD, but the physiological relevance of ERAD capacity in pancreatic β cells has been unclear. What is established is that SEL1L deficiency in β cells causes HRD1 instability (and, thus, hypofunction), and this leads to diminished islet insulin content (17), but the impact on β cell proinsulin content has been controversial (6). HRD1-mediated ERAD capacity for proinsulin might be exceeded in early type 2 diabetes when β cell proinsulin levels — and misfolded proinsulin levels — are unusually elevated, such as has been observed in the islets of young *db/db* mice (43, 49). Deficient ERAD capacity can be modeled in β cells bearing genetic or pharmacologic deficiency of HRD1. We find that rather than any loss of islet cell mass (17) or size (see Supplemental Figure 2), our findings strongly support the view that deficient β cell ERAD capacity results in a marked decrease of total islet proinsulin as well as insulin (Figure 2, C–E). Nevertheless, based on examination of *β-Hrd1*–KO mice as well as β cells acutely treated with HRD1 inhibitor, we propose that what is increased is the relative abundance of misfolded proinsulin (Figure 2B, Figure 6, B, D, and E, and Supplemental Figures 7–9), which tends to shift the distribution of intracellular proinsulin away from its normal juxtanuclear Golgi-like concentration with redistribution toward the ER (Figure 3A, Figure 7C, Supplemental Figure 4A, and Supplemental Figure 11).

The loss of β cell ERAD capacity triggers islet cell heterogeneity, including evidence of β cell dedifferentiation with an increase of ALDH3-positive cells (Figure 3 and Supplemental Figure 4B) (17), as has been observed in diabetic states from multiple proximal stresses (including defects that trigger chronically increased cytosolic calcium) — both related and unrelated to ERAD deficiency (17, 26, 27, 50–60). This, as well as the increased abundance of glucagon-positive cells within the islet interior (Figure 3C), may contribute to but may not solely be responsible for the development of diminished islet proinsulin levels, as the increased fraction of misfolded proinsulin in the ER of β cells with either genetically or pharmacologically impaired HRD1 is accompanied by increased phospho-eIF2α (Figure 5 and Figure 7C) that limits proinsulin biosynthesis, which lowers proinsulin levels (61). Although the *β-HRD1*–KO mice develop diabetes with abnormally small insulin secretory granules (Figures 1 and 4, and Supplemental Figure 5), the increase of phospho-eIF2α (Figure 5, A and B) seems unlikely to simply reflect elevated extracellular glucose (62) because it also occurs upon acute β cell HRD1 inhibition under conditions in which glucose is invariant (Figure 6, B and C, and Supplemental Figure 9), as well as in human islets (Supplemental Figure 8).

ERAD dysfunction can increase ER stress (63), and the increase of phospho-eIF2α is a cardinal feature of the integrated stress response (64) that includes PERK as a potential eIF2α kinase (65). Although we have not proven that PERK is responsible for increased phospho-eIF2α upon HRD1 inhibition, it is certainly a potential candidate, as we have also observed other features typical of ER stress response in the islets of *β-Hrd1*–KO mice, including a 3- to 6-fold increase of BiP and p58^ipk^ (Figure 5), whereas the increased levels of IRE1α and SEL1L (Supplemental Figure 1C) are more likely to be a direct consequence of diminished ERAD-mediated turnover of these proteins (23). Phospho-eIF2α is a potent suppressor of new proinsulin synthesis (36, 39, 66, 67), which is detected conveniently in a simple and novel assay (Supplemental Figure 10A and Figure 6F) (38, 44). Indeed, upon HRD1 inhibition, the increase of phospho-eIF2α corresponds to a clear block in proinsulin synthesis, and this is confirmed by metabolic labeling studies (Supplemental Figure 10B). Thus, diminished proinsulin synthesis is at least one potentially important contributor to a decrease of intracellular proinsulin levels in β cells with deficient ERAD capacity. The notion that β cell ER stress caused by ERAD deficiency may contribute to impaired β cell Ca^2+^ homeostasis, resulting in additional β cell defects, is a possibility that also deserves further investigation.

As an additional consideration, we find that acute inhibition of HRD1 function in a β cell line leads to activation (lipidation) of LC3b (Figure 6G), similar to that seen in human islets (Supplemental Figure 8), and upon chronic ERAD inhibition in the islets of *β-Hrd1*–KO mice (Figure 2B). Trapping autophagy flux in *β-Hrd1*–KO islets with lysosomal inhibitors — in addition to limiting the turnover of LC3b-II — results in accumulation of proinsulin (Figure 6A) in the form of aberrant disulfide-linked complexes (Figure 6B). The abnormal increase of misfolded proinsulin can be seen within 2 h of HRD1 inhibition; the major misfolded forms to appear are nonnative monomers and disulfide-linked complexes seen in β cell lines and human islets (Figure 7 and Supplemental Figures 7–9). These forms appear preferentially conveyed to lysosomes as they are accumulated by treatment with lysosomal inhibitors (Figure 6). While accumulation of disulfide-linked complexes of proinsulin is also observed upon bafilomycin treatment of islets from control animals (Figure 6A), the data suggest that in a state of diminished ERAD capacity, misfolded proinsulin is susceptible to accumulating in disulfide-linked complexes that can be transported via ER-phagy to lysosomes. Thus, along with suppressed proinsulin synthesis, increased autophagic flux is another key pathway that can account for diminished intracellular proinsulin levels in β cells that have exceeded their HRD1-mediated ERAD capacity.

We have recently reported that when ERAD capacity is exceeded, the IRE1α signaling pathway may also contribute to activation of β cell autophagy (19). Even more recent work indicates that expression of the ΣR1 gene product, which itself has been found to be an ERAD substrate (68), recruits the LC3b mRNA for LC3b-I translation at the ER, which is a precondition that favors its subsequent lipidation to LC3b-II (20). Upon ΣR1 KD in INS1 β cells, acute inhibition of ERAD cannot stimulate the same level of generation of LC3b-II (Figure 7, A and B) and limits the appearance of proinsulin puncta in forming autophagosomes (Figure 7C and Supplemental Figure 11). These findings help to further map the molecular crosstalk between ERAD and autophagic clearance pathways in β cells (17, 19), which in turn impacts proinsulin, and ultimately insulin, levels.

In conclusion, in a state of deficient ERAD capacity, the combined impact of a diminution of mature β cells, an increase in islet β cell phospho-eIF2α with diminished proinsulin synthesis, and enhanced autophagic clearance results in a diminished level of proinsulin that results in markedly decreased insulin content with impaired glucose tolerance (Figure 1, A–C). Indeed, in the setting of diminished insulin storage in β cells (Figure 4), serum insulin cannot keep up with demand (Supplemental Figure 3B), leading to diabetes (Figure 1E). Many additional gene products linked to ER quality control–dependent ubiquitin-proteasomal proteolysis also show genetic evidence of linkage to human type 2 diabetes (such as EDEM3, UFD1, NPL4, BAG1, and FAF1). It is plausible that some of these risk alleles, which act in concert with the SEL1L-HRD1 ERAD machinery, may contribute to a state of diminished ERAD capacity in β cells, potentially leading to versions of the dramatic phenotypes described in the current study.

## Methods

### Sex as a biological variable.

Animals of both sexes were analyzed and included in the study, as shown in all graphs within the manuscript (males = squares; females = circles); there were similar findings for both sexes. For analyses of glucose tolerance, our study design accounted for sex as a biological variable, as shown in Figure 1. Elsewhere, unless otherwise stated, results from both male and female animals were combined.

### Mice.

All mice were in a C57BL6/j background. *Hrd1*-floxed mice (69) and *RIP-Cre* mice (21) were used as previously described to generate β cell–specific *β-Hrd1*–KO mice. Control littermates used for experiments were β cell heterozygous deletion of *Hrd1*, also known as *Synv1*. Random blood glucose was measured weekly (and at the time of euthanasia) by a OneTouch Ultra blood glucometer and test strips. For intraperitoneal glucose tolerance tests, mice were fasted for 6 h, d-glucose (1 g/kg body weight) was administered intraperitoneally, and tail vein glucose was monitored (by a OneTouch Ultra glucometer) at different time points thereafter. Where indicated, circulating serum insulin levels were measured using the Mouse Ultrasensitive Insulin ELISA Kit (80-INSMSU-E10; ALPCO). Islet isolation was performed as described previously (49). For overnight recovery, islets were incubated in complete RPMI-1640 medium in a humidified 5% CO_2_ incubator at 37°C.

### Reagents and antibodies.

All reagents and chemicals were from Thermo Fisher Scientific or Sigma-Aldrich, except for the ER translocation inhibitor TL033 (Supplemental Figure 10), which was obtained from T.W. Bell (University of Nevada, Reno, Nevada, USA) (44). SDS-PAGE 4%–12% Bis-Tris or 12% Tris-Glycine NuPage gels were purchased from Thermo Fisher Scientific. Antibodies in this study included mouse mAb antirat proinsulin (CCI-17, Novus, RRID: AB_1107982), guinea pig anti-insulin (Covance, RRID: AB_10013624), rabbit anticyclophilin B (Thermo Fisher Scientific, RRID: AB_2169138), rabbit mAb anti-Hsp90 (Cell Signaling, RRID: AB_2233307), rabbit mAb anti-LC3A/B (Cell Signaling, RRID: AB_2617131), rabbit polyclonal anti-ALDH1A3 (Thermo Fisher Scientific, RRID: AB_2546664), mouse mAb antiglucagon (Abcam, RRID: AB_297642), rabbit polyclonal anticalnexin (Proteintech, RRID: AB_2069033), rabbit anti-IAPP (human residues 25–37, cross-reacting with rodent; BMA Biomedicals, catalog T-4157), rabbit mAb anti-p58^ipk^ (Cell Signaling, RRID: AB_2095213), rabbit mAb anti–phospho-eIF2α (Cell Signaling, RRID: AB_390740), rabbit mAb ΣR1 (Proteintech, RRID: AB_2301712), rabbit polyclonal anti-Hrd1 (Proteintech, RRID: AB_2287023), rabbit polyclonal anti-FIP200 (Proteintech, RRID: AB_10666428), rabbit mAb anti-IRE1α (Cell Signaling, RRID: AB_823545), and mouse mAb antihuman proinsulin Β-C junction sequence (Abmart, RRID: AB_2921300).

### Cell culture and ΣR1 KD.

INS1E (obtained from the laboratory of C. Wollheim, University of Geneva, Geneva, Switzerland) and INS/832/13 (obtained from the laboratory of C. Newgard, Duke University, Durham, North Carolina, USA) rat pancreatic β cells were cultured in RPMI-1640 medium (supplemented with 10% FBS, 10 mM HEPES, 1 mM sodium pyruvate, penicillin/streptomycin, and 0.05 mM β-mercaptoethanol). For LS102 treatment, cells were seeded into 12-well plates; and 24 or 48 h later, the cells were fed with fresh complete medium and treated for 2 h with vehicle or 20 μM LS102.

For ΣR1 KD, INS1E (or INS832/13) cells grown to 70% confluence in 6-well plates were transfected with RNAiMax (Invitrogen) containing ΣR1 siRNA (5′-GGCUUGAGCUCACCACCUA annealed with 5′-UAGGUGGUGAGCUCAAGCC) or negative control oligos (Qiagen, 1027281) at a concentration of 100 nM. At 24 h, the cells were fed fresh complete media, and 24 h thereafter, cells were treated with LS102 in fresh media, as described above.

### Human islets.

Nondiabetic human pancreatic islets (90% purity, 95% viability; confirmed COVID negative) were generated at Prodo Labs or the University of Michigan Human Islet Core Facility and treated with LS102 as described above: donor 1: 59-year-old female, BMI 28.9 (HbA1c 5.4%), cause of death = stroke; donor 2: 52-year-old female, BMI 28.4 (HbA1c 5.2%), cause of death = anoxic event; donor 3: 38-year-old male, BMI 35.7 (HbA1c 5.6%), cause of death = cardiac event/anoxia; donor 4: 37-year-old male, BMI 35.6 (HbA1c 5.4%), cause of death = anoxia (drug intoxication); donor 5: 66-year-old female, BMI 32.4 (HbA1c 5.5%), cause of death = stroke; donor 6: 68-year-old female, BMI 26.5 (HbA1c 5.3%), cause of death = stroke; donor 7: 62-year-old female, BMI 45.1 (HbA1c 5.4%), cause of death = stroke; donor 8: 69-year-old female, BMI 28.0 (HbA1c 5.5%), cause of death = stroke.

### Western blotting.

Cells or islets were lysed in RIPA buffer (25 mM Tris, pH 7.5, 100 nM NaCl, 1% Triton X-100, 0.2% deoxycholic acid, 0.1% SDS, and 10 mM EDTA containing a protease and phosphatase inhibitor cocktail). The collected lysates were spun at 12,000*g* at 4°C for 15 min, and the supernatant was stored at –80°C. The lysates (5–10 μg) were heated in gel loading buffer (LDS, Invitrogen) at 95°C for 5 min ± 200 mM DTT (for reduced or nonreduced samples, respectively) and resolved by 4%–12% or straight 12% NuPAGE gel. Nonreduced gels were then either untreated or treated with 100 mM DTT at 60°C for 10 min (43) before electrotransfer to nitrocellulose. The membrane was blocked with 5% BSA followed by primary antibody incubation (overnight at 4°C) and then HRP-conjugated secondary antibody incubation at room temperature for 30 min. Blots were developed using Bio-Rad Clarity or Amersham ECL reagent.

### Metabolic labeling of INS1E cells and mouse pancreatic islets.

Cells treated ± LS102 for 2 h or isolated from control and *HRD1-*KO littermates were washed in prewarmed Met/Cys-deficient RPMI medium (Sigma-Aldrich, R7513, ± LS102) and then pulse-labeled with ^35^S–amino acids (Trans^35^S label, Revvity Health) for 15 or 20 min at 37°C. The cells or islets were washed with ice-cold PBS containing 20 mM N-ethylmaleimide (NEM)and then lysed in RIPA buffer containing 2 mM NEM and a protease inhibitor cocktail (islets were sonicated in this buffer). Cell lysates (normalized to TCA-precipitable counts) were precleared with pansorbin and immunoprecipitated with anti-insulin antibodies and protein A–agarose (Sigma-Aldrich, 11134515001) overnight at 4°C. Immunoprecipitates were washed and analyzed by reducing 4%–12% gradient NuPAGE or Tris-tricine-urea-SDS-PAGE, fixed, dried, and examined by phosphorimaging. Bands were quantified with ImageJ software.

### Immunofluorescence and image analysis.

Paraffin sections of formaldehyde-fixed pancreas were deparaffinized with CitriSolv (Fisher Scientific, 04-355-121) and rehydrated in a decreasing graded series of ethanol followed by heating for antigen retrieval (BioLegend, 927901). Slides were washed with PBS, incubated in TBS blocking buffer containing 0.2% Triton X-100 and 3% BSA for 2 h, and incubated in primary antibody (in TBS plus 3% BSA and 0.2% Tween 20) overnight at 4°C. After washes, secondary antibody was incubated for 1 h at room temperature. Slides were washed 3 times with TBS/0.1% Tween 20 and mounted with antifade mounting medium-plus-DAPI (Vector Laboratories, H-1800).

INS-832/13 β cells grow as relatively flat cells and thus are favorable for immunofluorescence (46) compared with INS1E cells. INS832/13 cells grown on coverslips (in 12-well plates) ± ΣR1 KD, as described above, were either treated with LS102 or with vehicle (DMSO) for 90 or 120 min beginning at 48 h after siRNA transfection, then briefly washed in PBS, fixed in 4% formaldehyde (20 min at room temperature), washed twice in PBS, permeabilized in 100% cold methanol (10 min at –20°C), washed thrice in TBS, blocked in 3% BSA + 0.2% Tween-20 in TBS (1 h at room temperature), incubated with primary antibody (1:500) in the same blocking buffer (overnight at 4°C), washed thrice again in TBS, incubated with secondary antibody (1:500) in blocking buffer (1 h at room temperature), and finally washed thrice in TBS. Coverslips were placed on a drop of Prolong Gold antifade reagent plus DAPI and mounted on glass slides. For both tissue and cell culture, epifluorescence was imaged on a Nikon A1 confocal microscope, with digital images analyzed by NIS-Elements/ImageJ software.

### Transmission electron microscopy.

Islets freshly isolated from control or *β-Hrd1*–KO mice were fixed in 2.5% glutaraldehyde prepared in 0.1 M sodium cacodylate buffer (pH 7.2), embedded in low-melting-point agarose, cut into approximately 1 mm cubes, postfixed in osmium tetroxide/potassium ferrocyanide, and washed sequentially in sodium cacodylate buffer followed by 0.1 M sodium acetate buffer. The cubes were stained in uranyl acetate (in sodium acetate buffer), dehydrated through a graded ethanol/acetone series, and embedded in Spurr’s resin. Ultrathin sections were examined with a transmission electron microscope (JEOL USA; at 80 kV) and digital camera.

### Statistics.

To assess statistical differences between 2 groups, we employed a 2-way ANOVA with Šidák’s multiple-comparison test for data built on 2 independent variables (Figure 1 and Supplemental Figure 3, A and B) or 2-tailed Mann-Whitney *U* test (Supplemental Figure 1D, Supplemental Figure 2, and Supplemental Figure 5). All other statistical analyses used an unpaired 2-tailed *t* test for data showing a normal distribution. In each case, the data are presented as the mean ± SD; a *P* value less than 0.05 was considered statistically significant.

### Study approval.

All animal procedures were approved by and done in accordance with the IACUC at the University of Michigan Medical School (PRO00011324).

### Data availability.

All data are contained within the figures; additionally, quantitation presented in the figures is documented the Supporting Data Values file. Reagents are available upon request from the corresponding author.

## Author contributions

AA, BT, LQ, and PA designed research studies. AA, LH, MA, NFG, EM, SB, JK, CYC, IM, and AH conducted experiments and acquired and analyzed data. DL and DF provided key reagents. PA wrote the manuscript. All authors reviewed, edited, and approved the manuscript.

## Funding support

This work is the result of NIH funding and is subject to the NIH Public Access Policy. Through acceptance of this federal funding, the NIH has been given a right to make the work publicly available in PubMed Central.

- NIH grant R01DK143292 (to BT, LQ, and PA).
- NIH grant R01DK48280 (to PA).

## Supplementary Material

Supplemental data

Unedited blot and gel images

Supporting data values

## Version 1 11/18/2025

In-Press Preview

## Version 2 01/16/2026

Electronic publication
