# Supplementary material for: Limiting ER-associated degradation capacity triggers acute and chronic effects on insulin biosynthesis
Source: J Clin Invest. 2025 Nov 18;136(2):e187341. doi: 10.1172/JCI187341 (PMC12807472; doi:10.1172/JCI187341)
Supplement: Supplemental data [file jci-136-187341-s012.pdf]

Supplement

Figures

Arunagiri et al.

Supplemental Figure Legends

Figure S1. *Ins1Cre*-medated *Hrd1* KO in pancreatic β-cells. (A)) Isolated islets from a 4-5 week-old *β-Hrd1-KO* or control (random blood glucose values shown at top): immunoblotting for insulin (n = 4); HSP90 is a loading control. (B) Insulin content (by ELISA) in islets isolated from control or *β-Hrd1-KO* mice (mean age and blood glucose indicated; n=4 per group; unpaired two-tailed *t*-test ***p*<0.01). (C) Islet lysates from a 5-week-old *β-Hrd1-KO* and littermate control (random blood glucose values shown at top) immunoblotted at left for IRE1α (and loading control) and at right for SEL1L (and loading control). Graph above indicates SEL1L protein level in islets of *β-Hrd1-KO* mice normalized to that in control mice (n=4 animals per group; unpaired two-tailed *t*-test **p*<0.05). (D) Glucose-stimulated insulin secretion in islets isolated from control (4 males, mean random blood glucose 138 mg/dL) or *β-Hrd1-KO* mice (3 males + 1 female, mean random blood glucose 219 mg/dL).

Figure S2. Hematoxylin and Eosin (H&E) staining of pancreatic tissue sections from *β-Hrd1-KO* or control mice. At *left*: Representative images from control and *β-Hrd1-KO* mice were examined for islet size (measured by ImageJ from fixed magnification images of random pancreas sections from each animal; scale bar = 200 μm). At *right*: islet areas quantified from n=3 mice per group (mean random blood glucose shown at bottom; no significant difference in islet size between groups by non-parametric two-tailed Mann-Whitney U test).

Figure S3. Body weight, serum insulin/glucose ratio, and islet IAPP content in *β-Hrd1-KO* mice. (A) Body weight measurements in control and KO mice (n = 8 per group; mean ± s.d; two-way ANOVA with Sidak's multiple comparison test; **p*<0.05). (B) Serum insulin/glucose ratio in cohorts of control or *β-Hrd1-KO* mice (in two different age groups representing diabetes progression; individual random blood glucose value indicated at each point and a "Hi" value was taken as 600 mg/dL; n = 3-7 per group mean ± s.d; two-way ANOVA with Sidak's multiple comparison test; ****p*<0.001). (C) Isolated islets from *β-Hrd1-KO* mice were analyzed by Western blot for IAPP content per ß-actin normalized to that seen from control mice; n=6 animals per group; both groups 4 males + 2 females; mean age and random blood glucose is shown; unpaired two-tailed *t*-test; ****p*<0.001).

Figure S4. Indirect immunofluorescence of proinsulin, calnexin, insulin, and ALDH1A3 in *β-Hrd1-KO* or control mice. (A) Pancreas sections from control or *β-Hrd1-KO* mice (n = 4 per group) were co-labeled with mAb anti-proinsulin (CCI-17, in red) and mAb anti-calnexin (in green) using quenching and sequential labeling. (B) Pancreas section of a *β-Hrd1-KO* mouse (random blood glucose indicated, representative of n = 7 animals of this genotype) with immunofluorescence for insulin (green), proinsulin (CCI-17, red) and

ALDH1A3 (blue). The boxed cells highlight beta-cells positive cells for insulin/proinsulin that are also positive for ALDH1A3.

Figure S5. Secretory granule diameters from control and *β-Hrd1-KO* mice. Transmission electron micrographs from each pair of animals (n=3 pairs of animals; 2 males + 1 female in each group), each measured at identical magnification. For each granule, the largest granule diameter was recorded; two-tailed Mann-Whitney U test, ****p<0.0001).

Figure S6. Side-by-side comparison of biosynthesis and steady-state level of proinsulin in islets from control and *β-Hrd1-KO* mice (females, 36 days old). (A) Isolated islets were pulse-labeled with ^{35}S-amino acids for 30 min and then chased for 2 h, as indicated. Cells ("C") and chase media ("M") were immunoprecipitated with anti-insulin (normalized to trichloroacetic acid–precipitable counts in the cell lysates), followed by Tris-tricine-urea-SDS-PAGE under nonreducing ("NR) or reducing ("R") conditions and phosphorimaging. (B) Additional islets from the same mice (random blood glucose listed above) were lysed in RIPA buffer and analyzed by reducing SDS-PAGE and immunoblotting with mAb anti-proinsulin; cyclophilin B (CypB) is a loading control.

Figure S7. Misfolded proinsulin increases after LS102 treatment. (A) INS1E cells were treated for 2 h with different concentrations (in μM) of LS102, and cell lysates were resolved by 4-12% NuPAGE under nonreducing or reducing conditions followed by proinsulin immunoblotting. The red arrows on the nonreducing side indicate non-native proinsulin dimers and trimers (marked D and T respectively), as well as higher molecular weight complexes. Beta-actin is a loading control. (B) INS1E cells were treated ± 20 μM LS102 x 2 h. Media were collected and cells were lysed; both were resolved by 12% NuPAGE under reducing or nonreducing conditions. The nonreduced gel then underwent post-gel disulfide reduction (with DTT + heating) before electrotransfer for anti-proinsulin immunoblotting — the green arrow indicates the native monomer and the red arrows indicate non-native monomer ("M"), and disulfide-linked dimer ("D"). This experiment was repeated in 6 biological replicates, with a doubling of nonnative dimer (**p = 0.005) and an 18% decrease of native proinsulin monomer (*p = 0.039) (C) Treatment of isolated human islets ± 20 μM LS102 x 2 h, followed by Western blotting for human proinsulin under nonreducing conditions followed by post-gel disulfide reduction to detect misfolded monomers and dimers (red arrows); LC3b shown below; HSP90 is a loading control.

Figure S8. Proinsulin misfolding in human islets after acute HRD1 inhibition. Human islets were treated as in Figure S7C but anti-proinsulin immunoblotting after nonreducing SDS-PAGE was without post-gel disulfide reduction, which enhances sensitivity of detection of aberrant proinsulin disulfide-linked complexes. (A) Immunoblotting (reducing SDS-PAGE) of human islet proinsulin, LC3, and phospho-eIF2α (vinculin is

a loading control). (B) Immunoblotting of human islet proinsulin after nonreducing SDS-PAGE. Quantitation of human islet (C) total proinsulin; (D) proinsulin misfolding; E) LC3-II/LC3-I; and (F) phospho-eIF2α. 5-7 per group; n numbers shown below each panel; unpaired two-tailed *t*-test, *p<0.05.

Figure S9. Increased misfolding of proinsulin newly-made in the presence of LS102 treatment. INS1E cells were treated as follows. The first well (lanes 1 and 5) was lysed at time 0h. The second well (lanes 2 and 6) was treated with cycloheximide (CHX, 100 µg/mL). The third and fourth wells were treated with CHX followed by washout (two washes in PBS) to promote recovery of protein synthesis for an additional 2 h in complete medium containing either vehicle (DMSO, lanes 3 and 7) or 20 µM LS102 (lanes 4 and 8). Cells were lysed and resolved by 12% NuPAGE under nonreducing (lanes 1-4) or reducing (lanes 5-8) conditions. The nonreducing gel underwent post-gel disulfide reduction before electrotransfer for immunoblotting with anti-proinsulin (upper panels) or anti-phospho-eIF2α (middle panels). The green arrow indicates native proinsulin; red arrows highlight a small amount of non-native monomers and disulfide-linked proinsulin dimers. HSP90 is a loading control (bottom panels). The data are representative of n=3 repeat experiments.

Figure S10. LS102 treatment affects proinsulin biosynthesis. (A) Immunoblotting of INS1E cells treated for 2h ± LS102 and additionally treated with TL033 (ER translocation inhibitor) + MG132 (proteasome inhibitor) during the last 30 min; appearance of the preproinsulin band can be used as a measure of proinsulin synthesis. Synthesis is inhibited ~60% by LS102 treatment (lanes 4 and 6 compared to 3 and 5). (B) INS1E cells treated for 2 h ± LS102 were pulse-labeled with ^{35}S-amino acids for 15 min (normalized to TCA-precipitable cpm) and immunoprecipitated with anti-insulin antibody (quantitation from n = 3 independent biological replicates shown at *left*; unpaired two-tailed *t*-test, *p<0.05; phosphorimage shown at *right*).

Figure S11. Proinsulin puncta induced by LS102 contain the ER marker, calnexin. INS832-13 cells were transfected with control oligo or ΣR1-KD, and after 48 h were treated ± LS102 (20 µM) for 2 h before fixation, permeabilization, and immunofluorescence for proinsulin (green) and the ER marker, calnexin (red): n = 6 independent biological replicates per group. Scale bar = 50 µm (all images).

Figure S12. Lysosomal inhibitors cause accumulation of misfolded disulfide-linked proinsulin complexes. (A) Nondiabetic human islets treated ± chloroquine (50 µM, 6 h) and analyzed by immunoblotting for proinsulin under nonreducing and reducing conditions (vinculin is a loading control). (B) After treatment of human islets as in (A), proinsulin disulfide linked-dimer / monomer ratio (an indicator of misfolding) is quantified; n = 5 per group; unpaired two-tailed *t*-test *p < 0.05. (C) After treatment of human islets as in (A), LC3-II is quantified (n = 4 per group; unpaired two-tailed *t*-test *p < 0.05). (D) *β-Hrd1-KO* mouse islets treated with chloroquine (50 µM, 24 h) and analyzed by immunoblotting for proinsulin under

nonreducing conditions; LC3 and CypB (loading control) are shown below. (E) Control or *β-Hrd1-KO* mouse islets treated with lysosomal inhibitor bafilomycin A1 (BafA1, 0.1 μM, 18 h) before analysis as in panel D (n = 2 independent biological replicates in panels D and E). Lysosomal inhibitors cause aberrant disulfide-linked proinsulin complexes to accumulate, and LC3-II also accumulates.

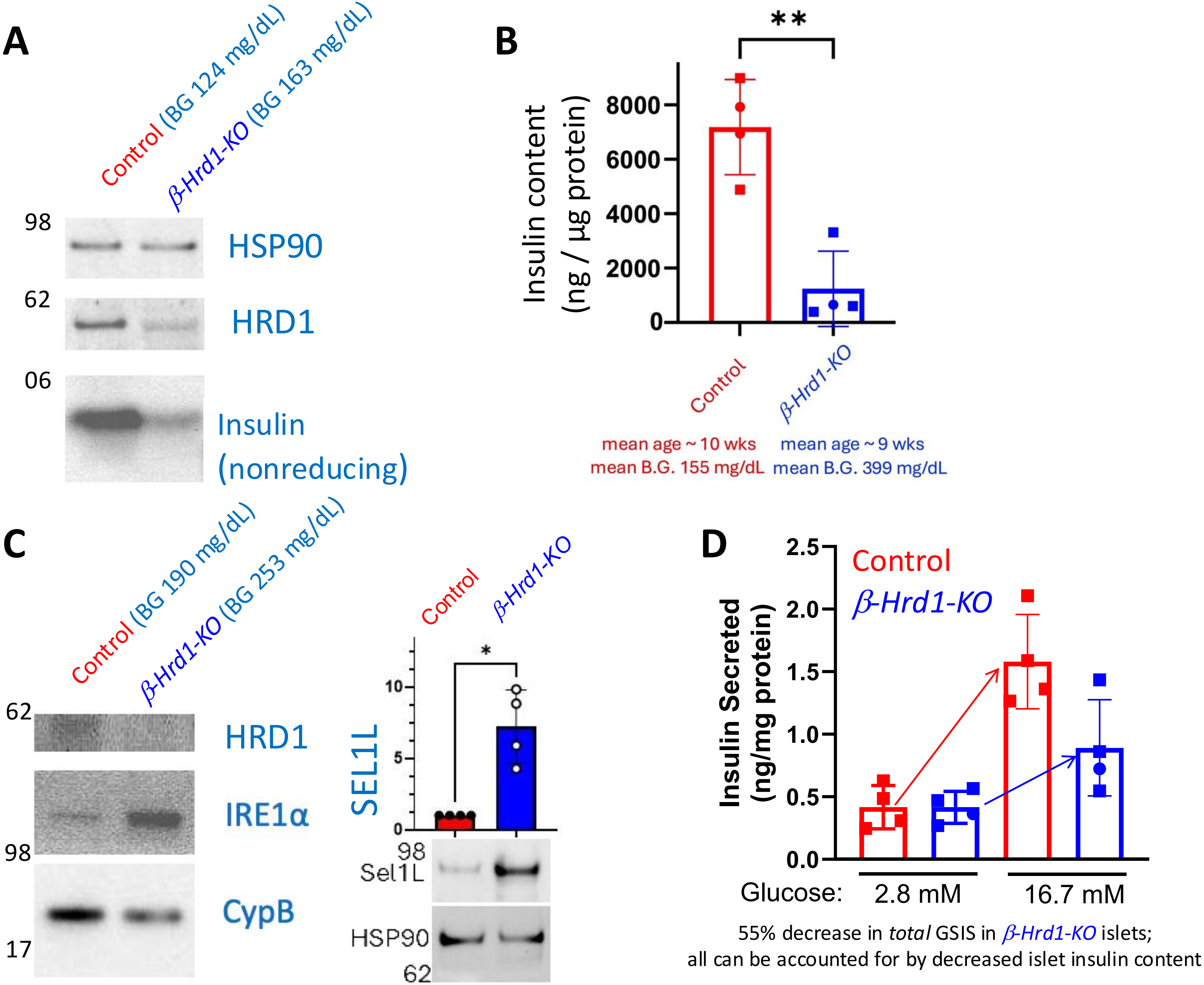

Arunagiri et al., Supplemental Fig. S1

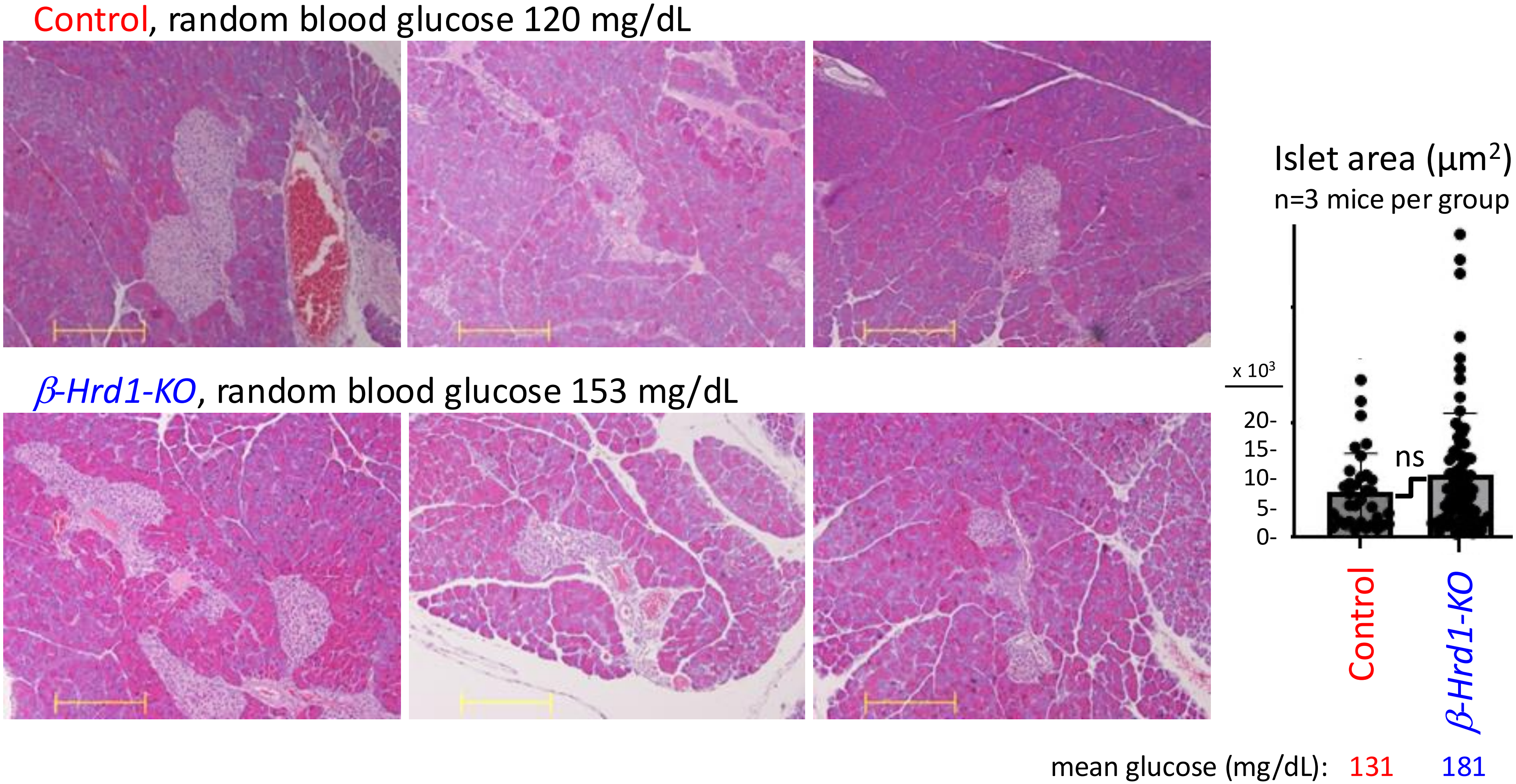

Arunagiri et al., Supplemental Fig. S2

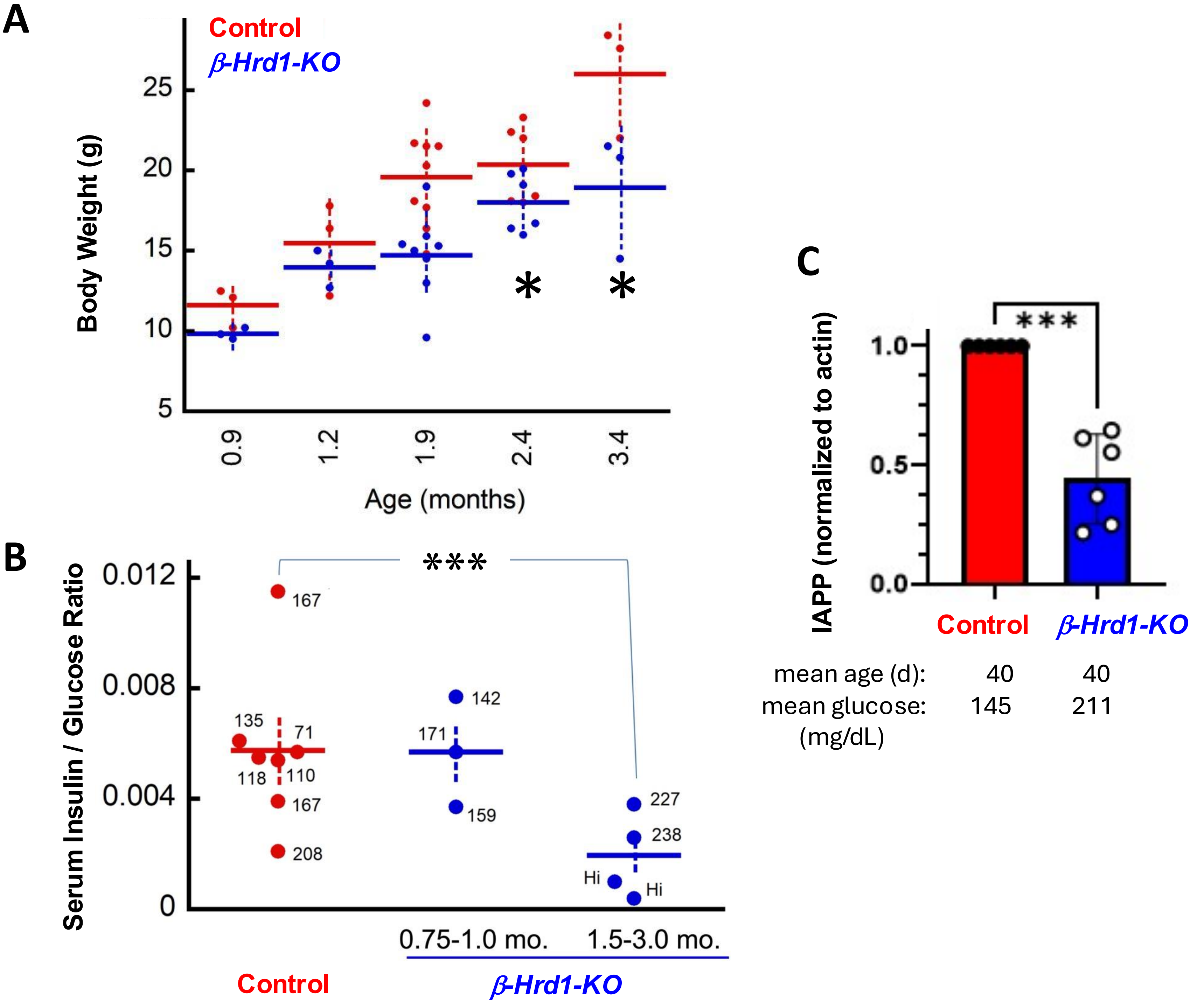

Arunagiri et al., Supplemental Fig. S3

A

Control
random blood glucose 139 mg/dL

β-Hrd1-KO
random blood glucose 153 mg/dL

B

β-Hrd1-KO
random blood glucose 355 mg/dL

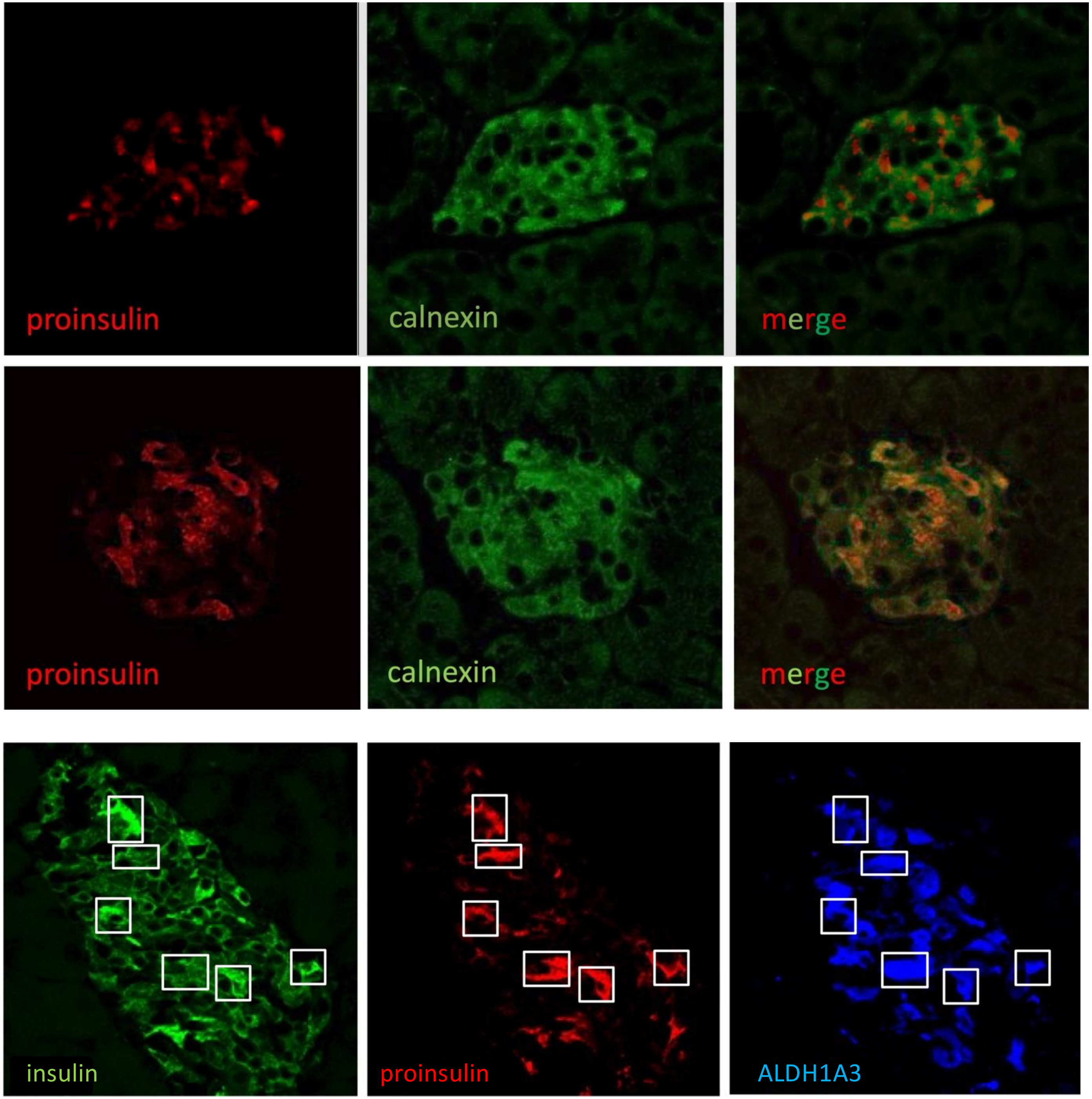

Arunagiri et al., Supplemental Fig. S4

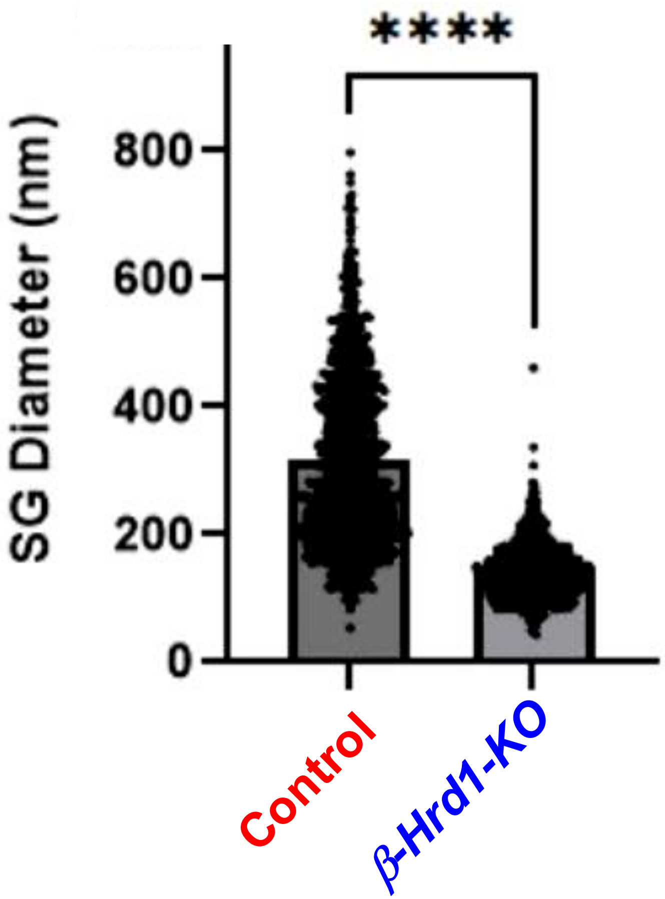

n = 3 mice per group

mean diameter
in ***β-Hrd1-KO***
is 45% of control

for circular geometry,
average granule
area in ***β-Hrd1-KO***
calculates to 20% of control

for spherical geometry,
average granule volume
in ***β-Hrd1-KO*** calculates
to 9% of control

Arunagiri et al., Supplemental Fig. S5

A

Pulse-chase analysis

mouse	Control			*β-Hrd1-KO*				Control			*β-Hrd1-KO*		
Cells (C) or media (M):	C	C	M	C	C	M		C	C	M	C	C	M
Chase time (h)	0	2	2	0	2	2		0	2	2	0	2	2
NR or R gel	NR	NR	NR	NR	NR	NR		R	R	R	R	R	R

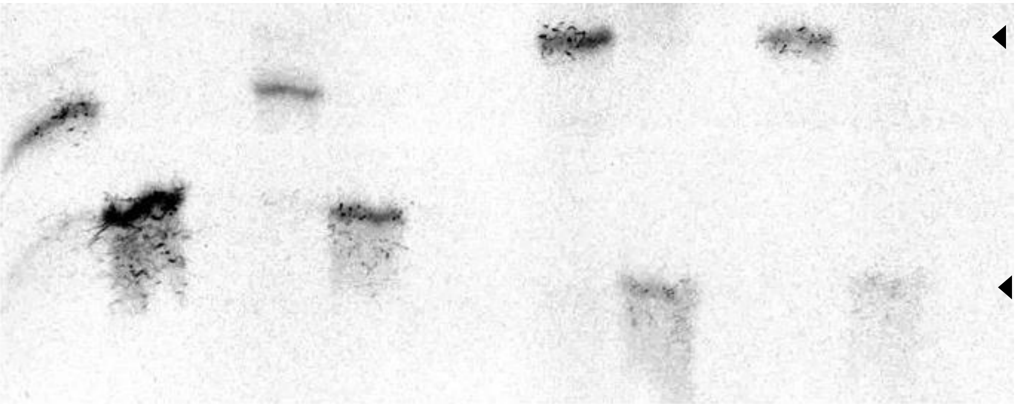

B

Immunoblotting

	Ctrl	KO
Glucose:	155	292

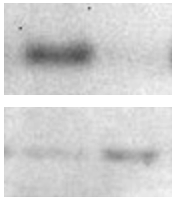

Decreased in steady-state proinsulin LEVEL is disproportionate to the decrease of pro-insulin biosynthesis in islets from *β-Hrd1-KO*.

Arunagiri et al., Supplemental Fig. S6

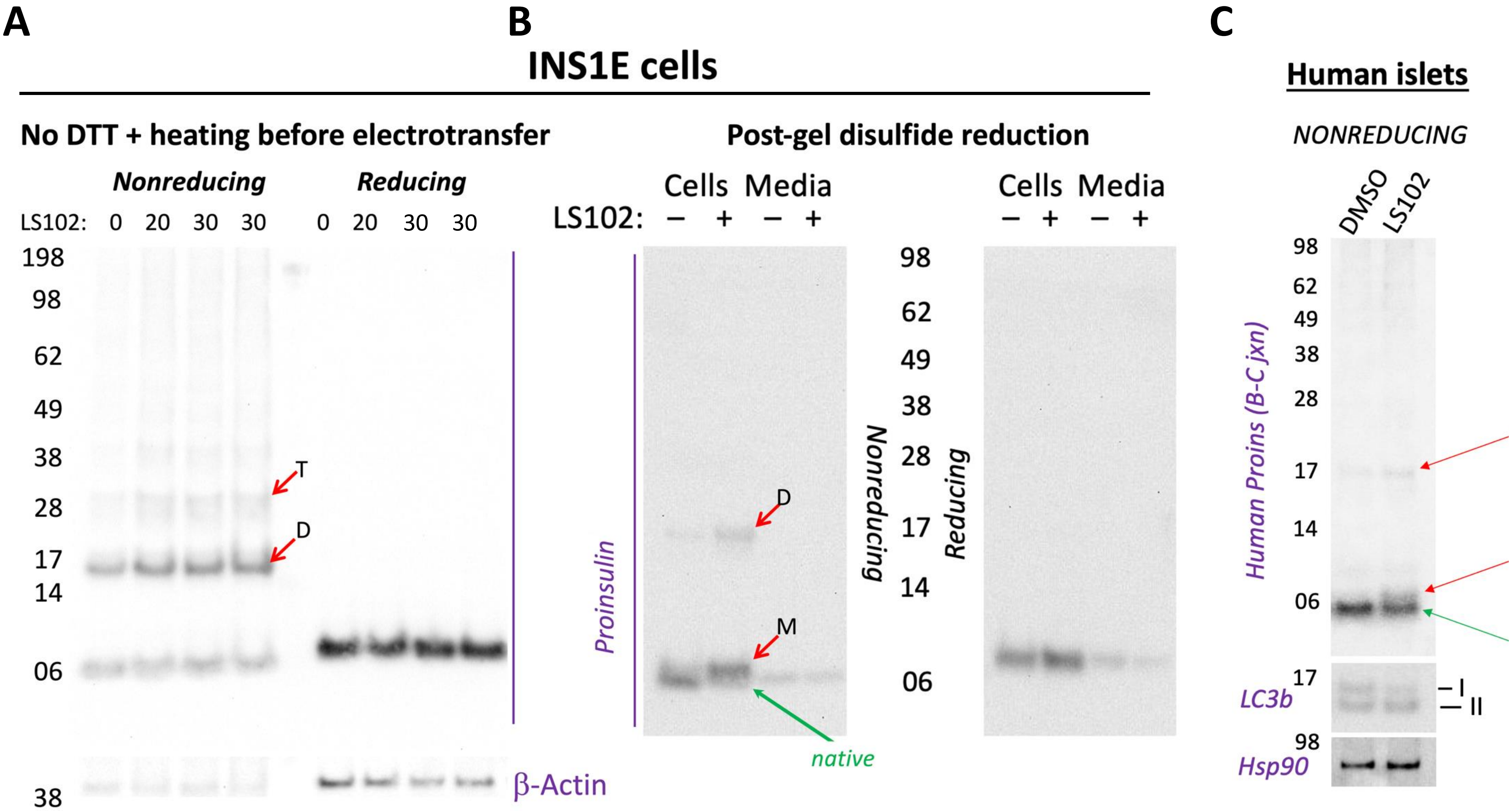

Arunagiri et al., Supplemental Fig. S7

Human Islets

± 20 μM LS102 x 2h; 5.5 mM Glucose

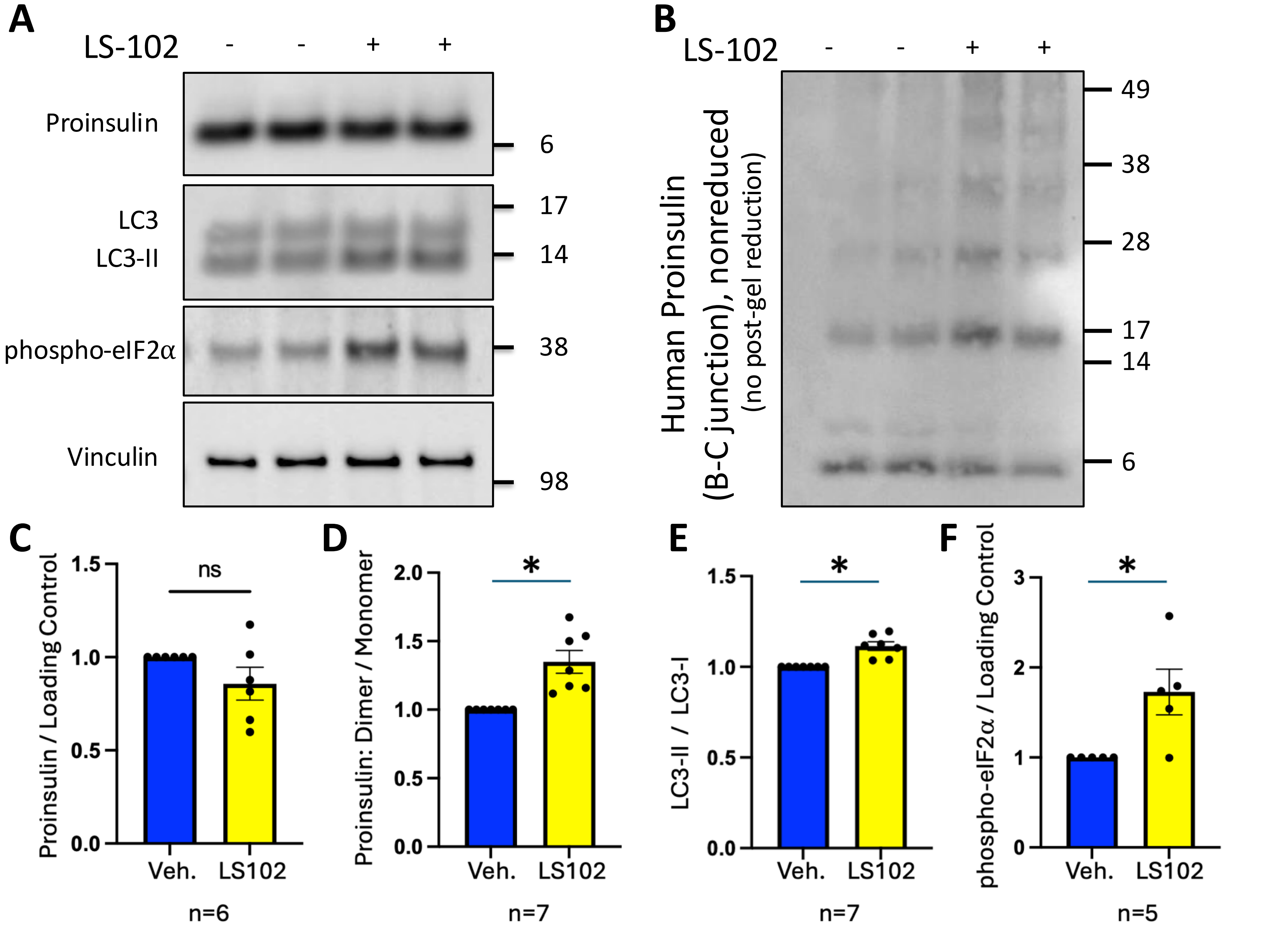

Arunagiri et al., Supplemental Fig. S8

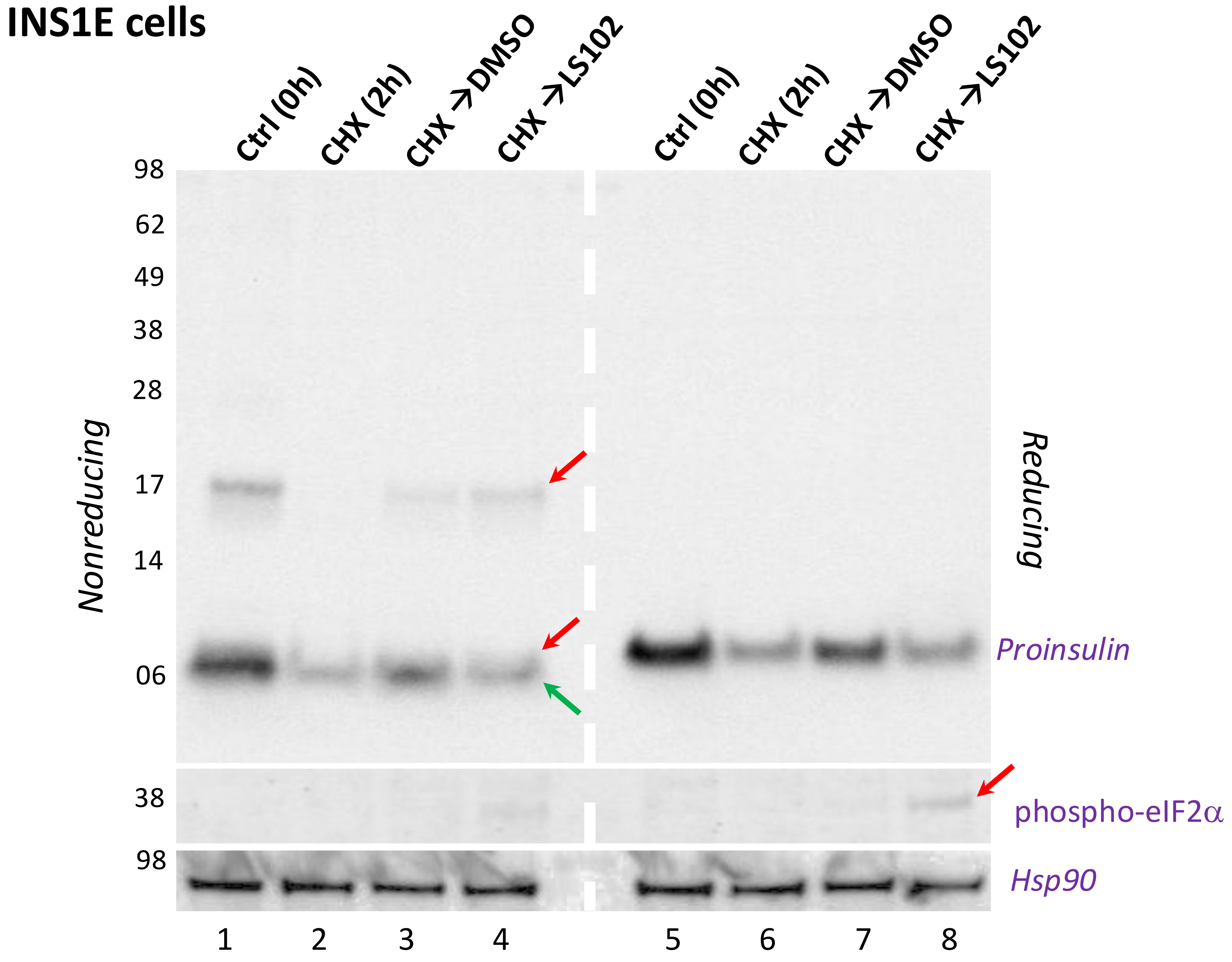

Arunagiri et al., Supplemental Fig. S9

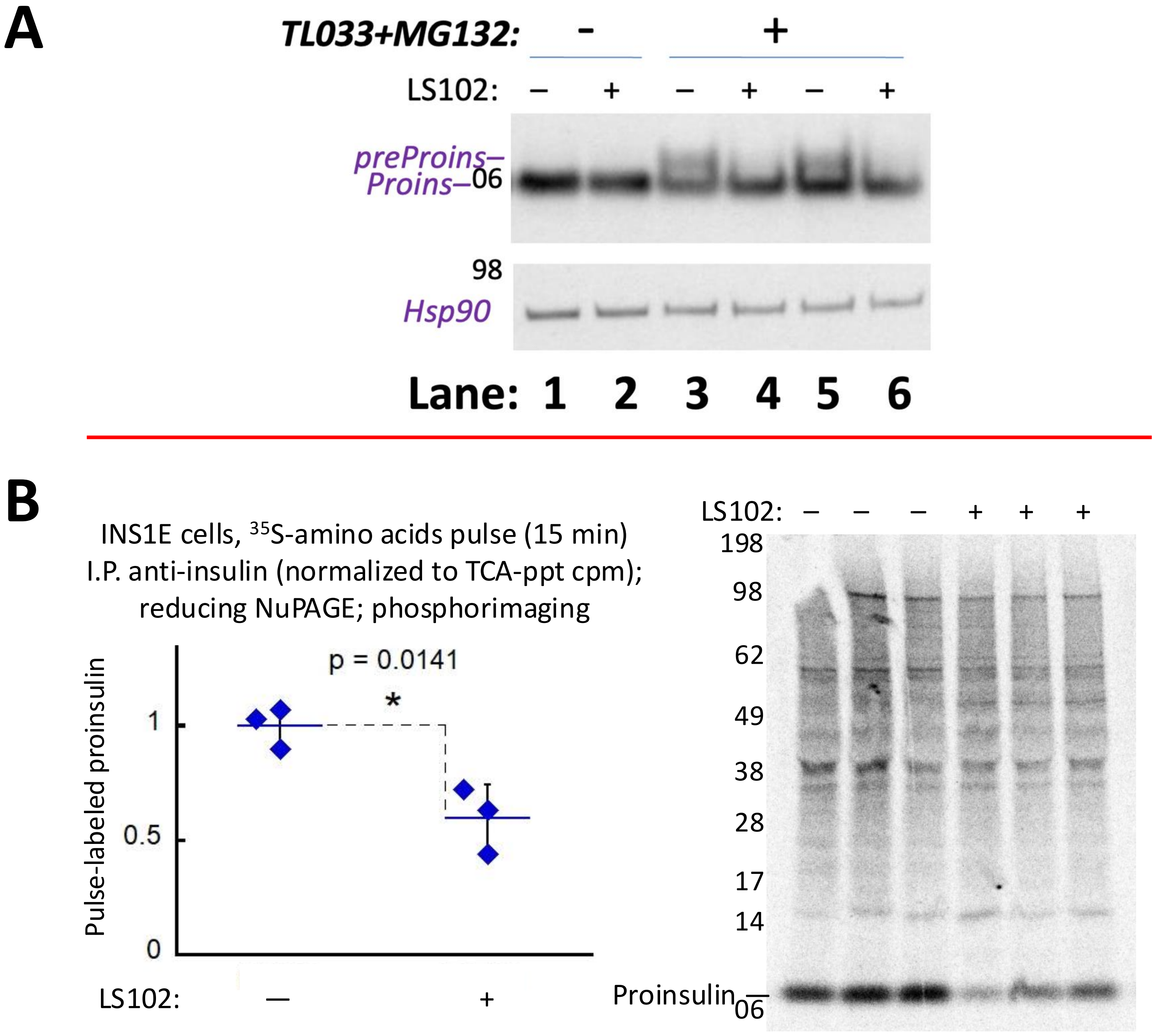

Arunagiri et al., Supplemental Fig. S10

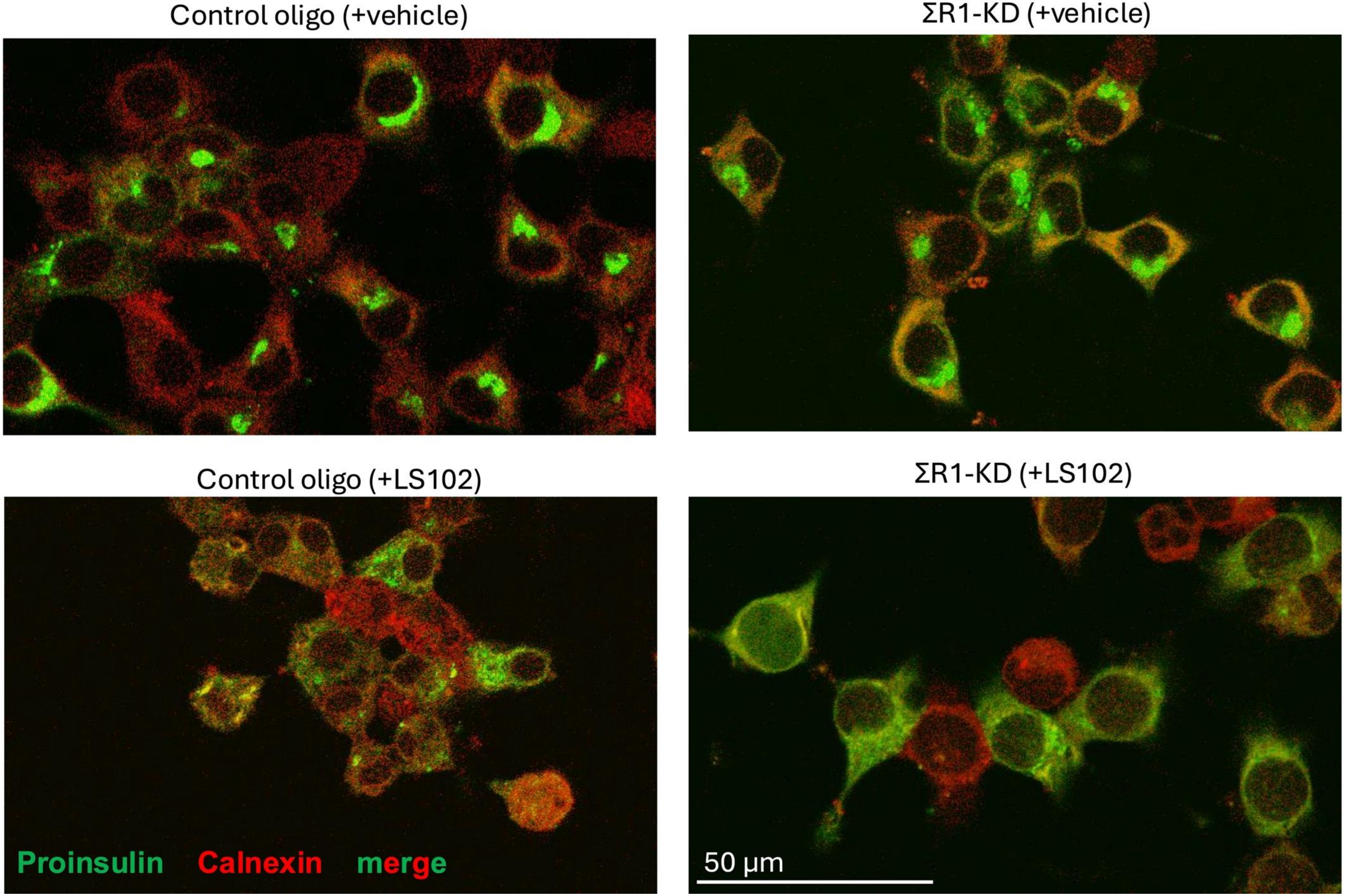

Arunagiri et al., Supplemental Fig. S11

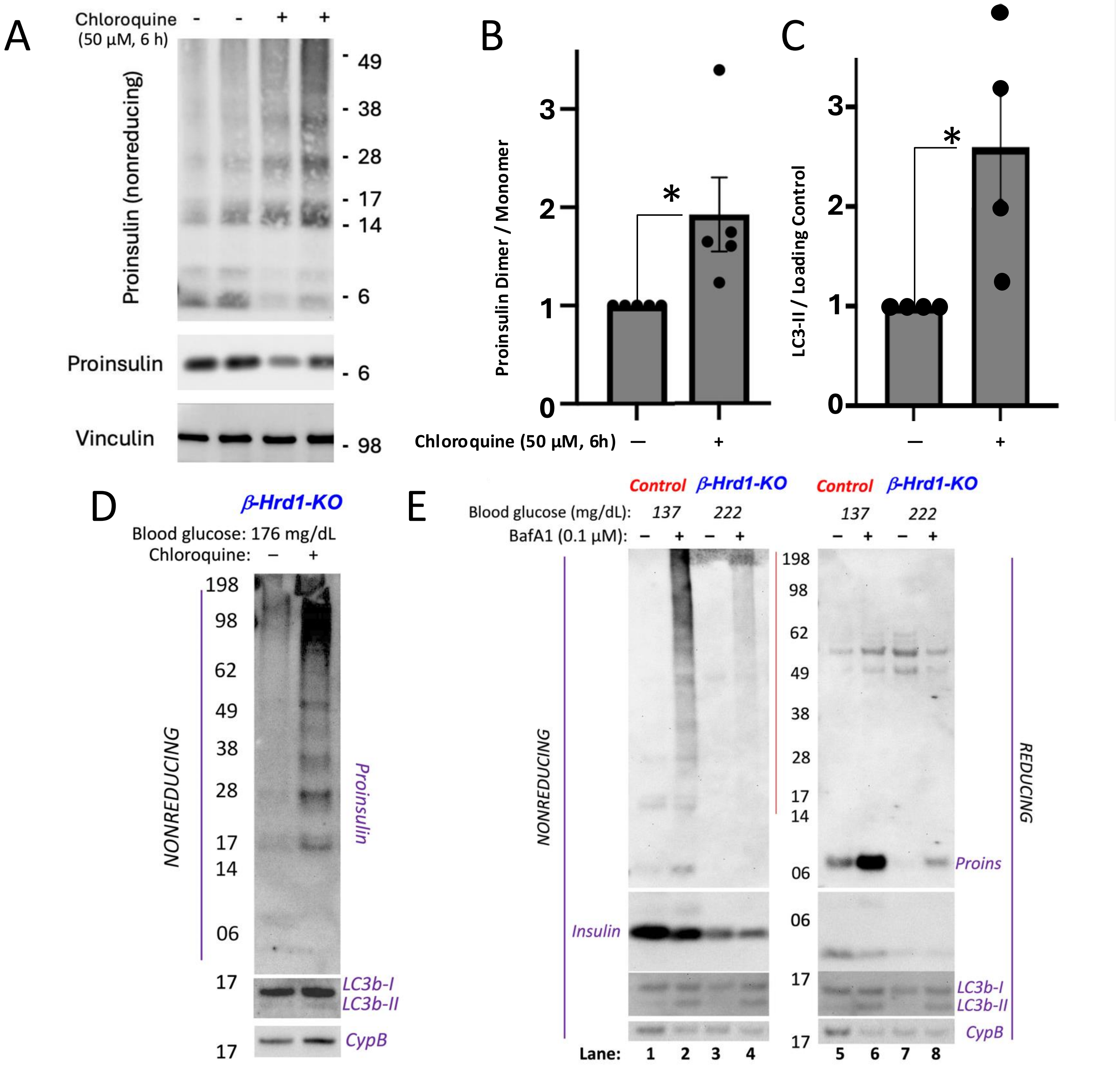

Arunagiri et al., Supplemental Fig. S12
