# Supplementary material for: Limiting ER-associated degradation capacity triggers acute and chronic effects on insulin biosynthesis
Source: J Clin Invest. 2025 Nov 18;136(2):e187341. doi: 10.1172/JCI187341 (PMC12807472; doi:10.1172/JCI187341)

Figure 2A

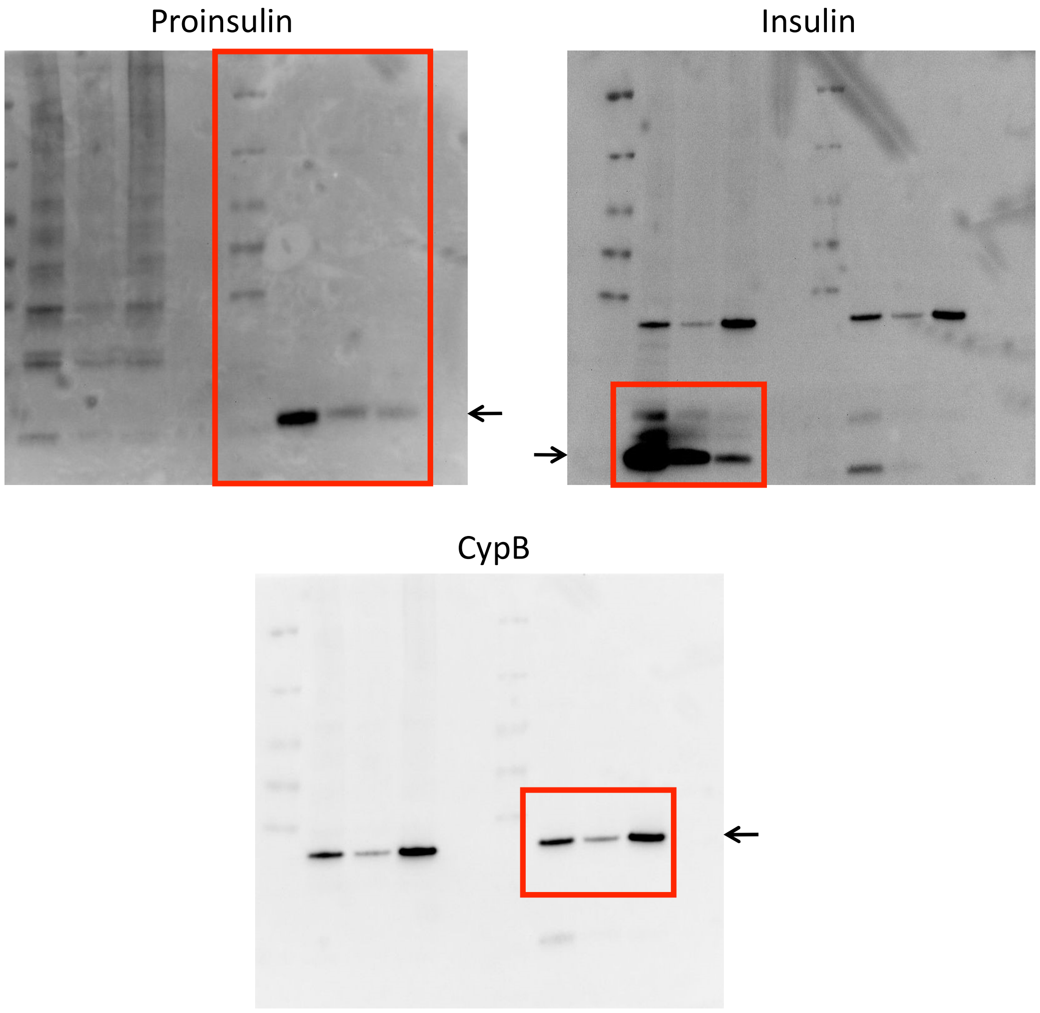

Figure 2B

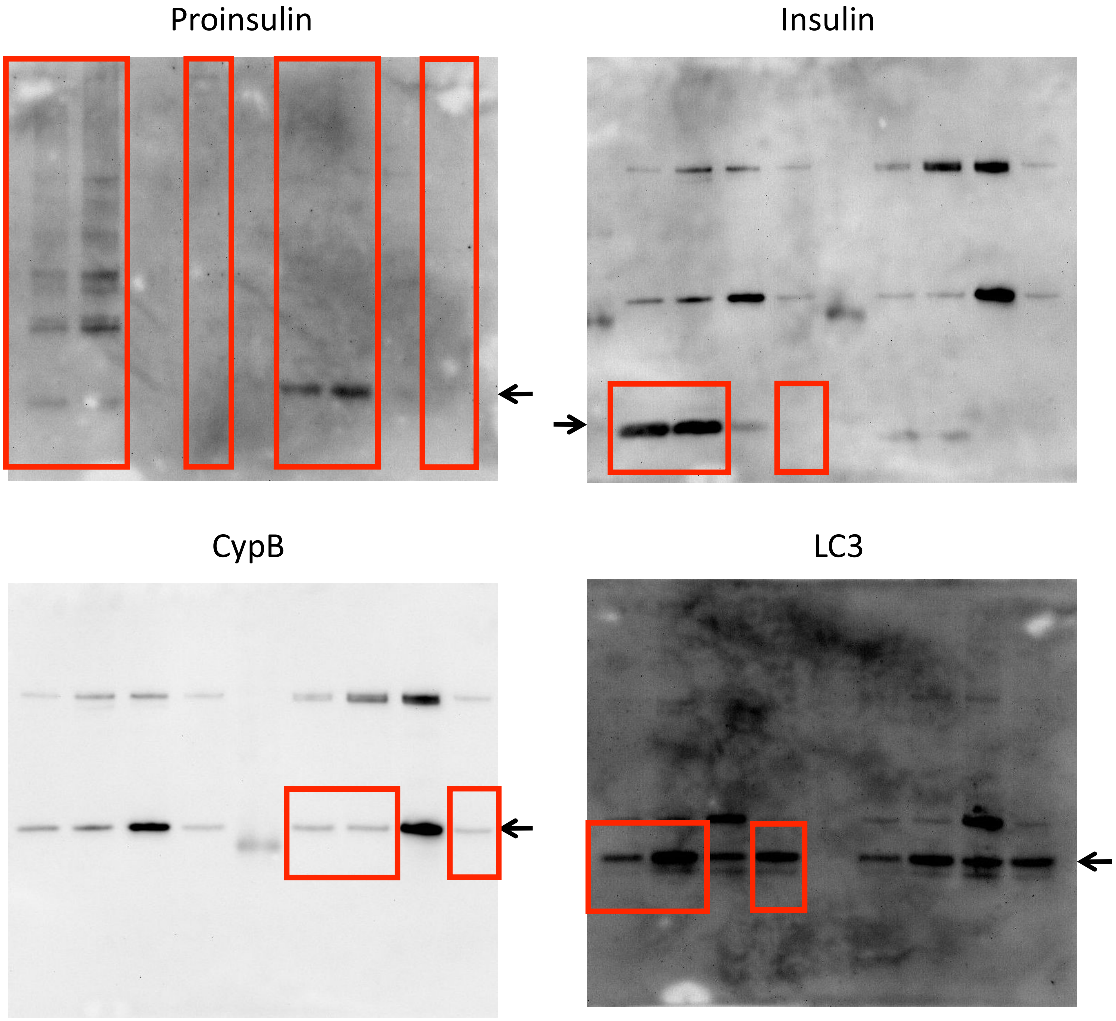

Figure 5
Upper
panel

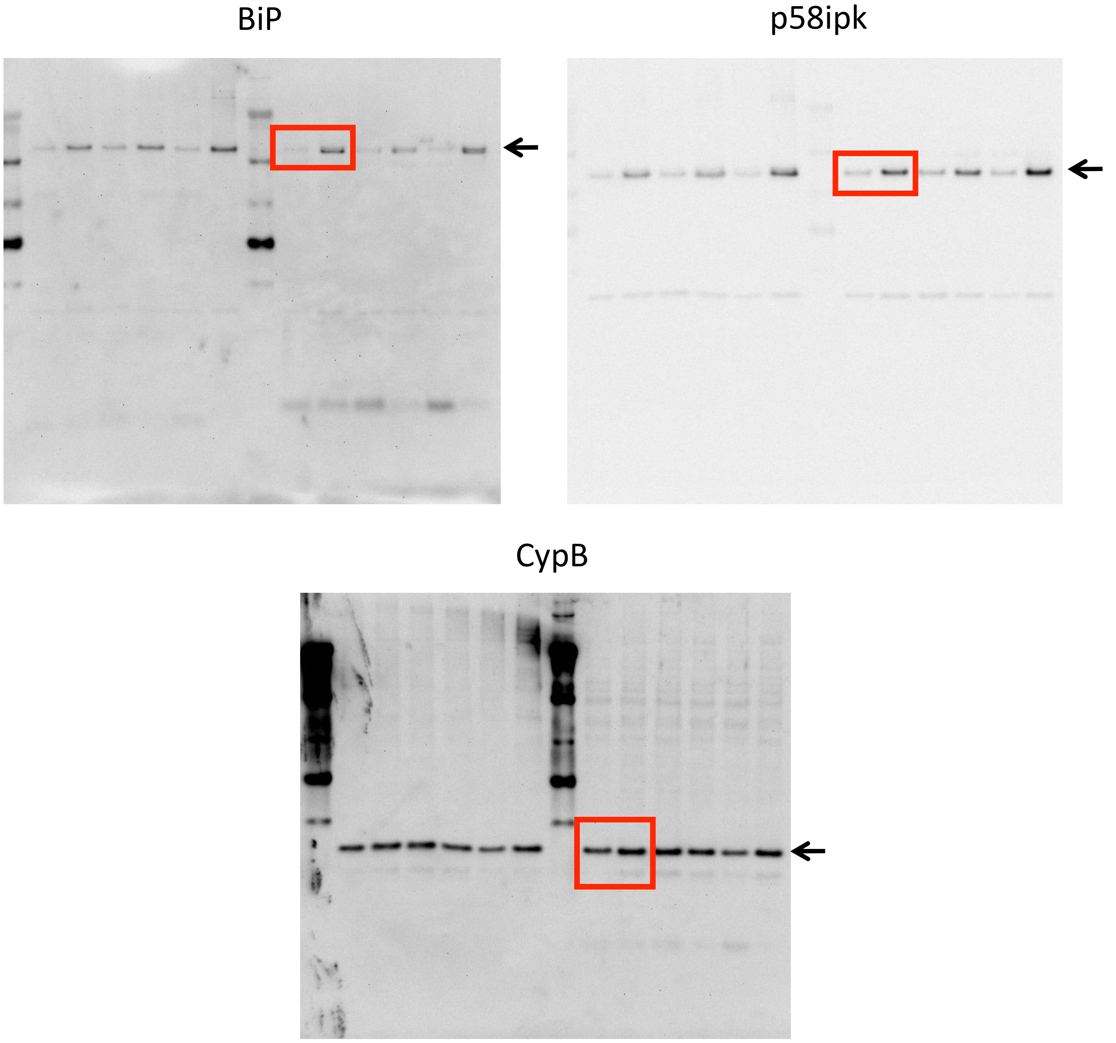

Figure 5
Lower
panel

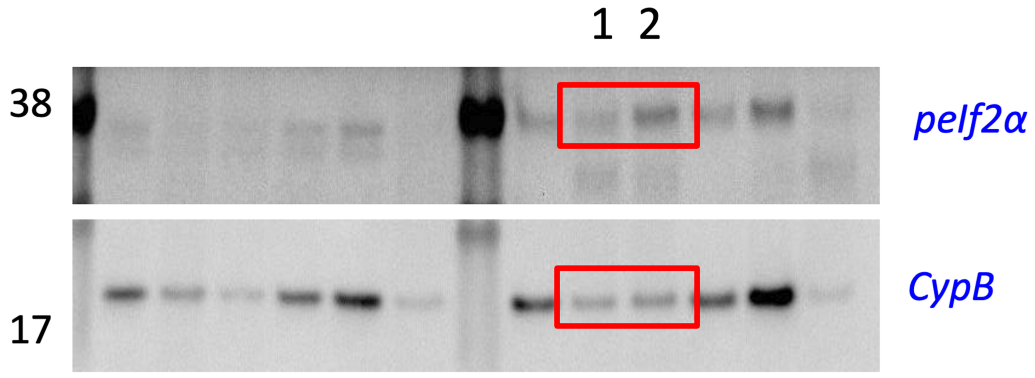

Replicate Experiment

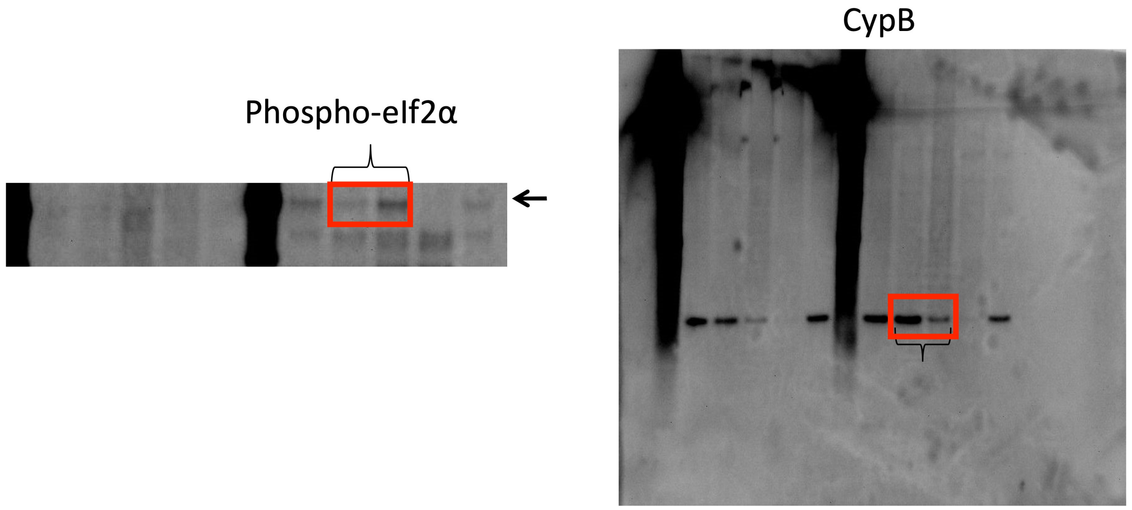

Third Experiment

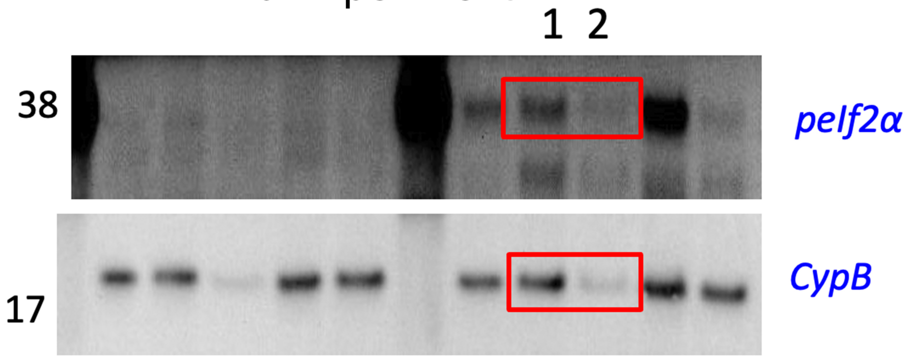

1: Hrd1flox/+ Cre
2: Hrd1KO, normoglycemic

Figure 6

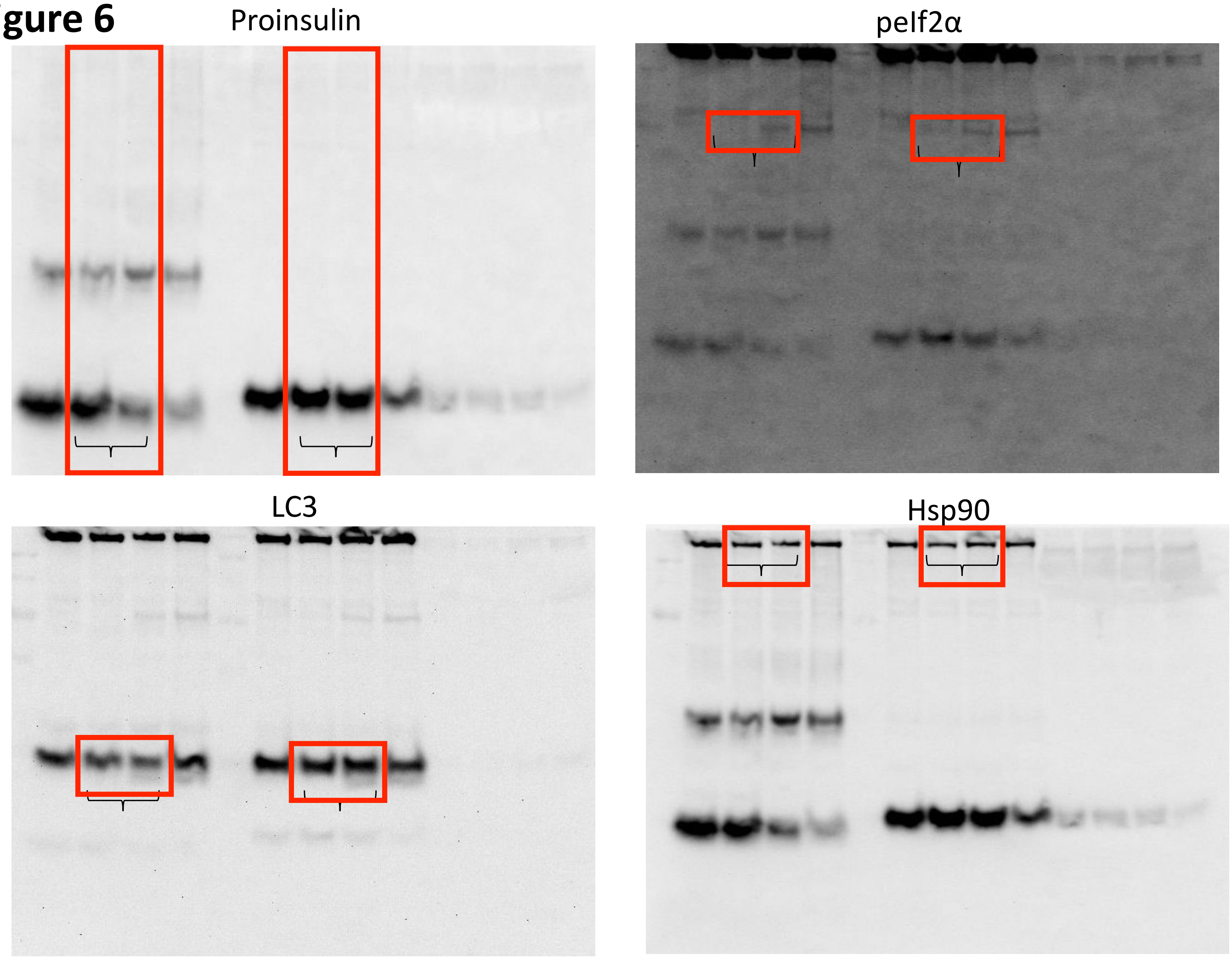

Figure 7A

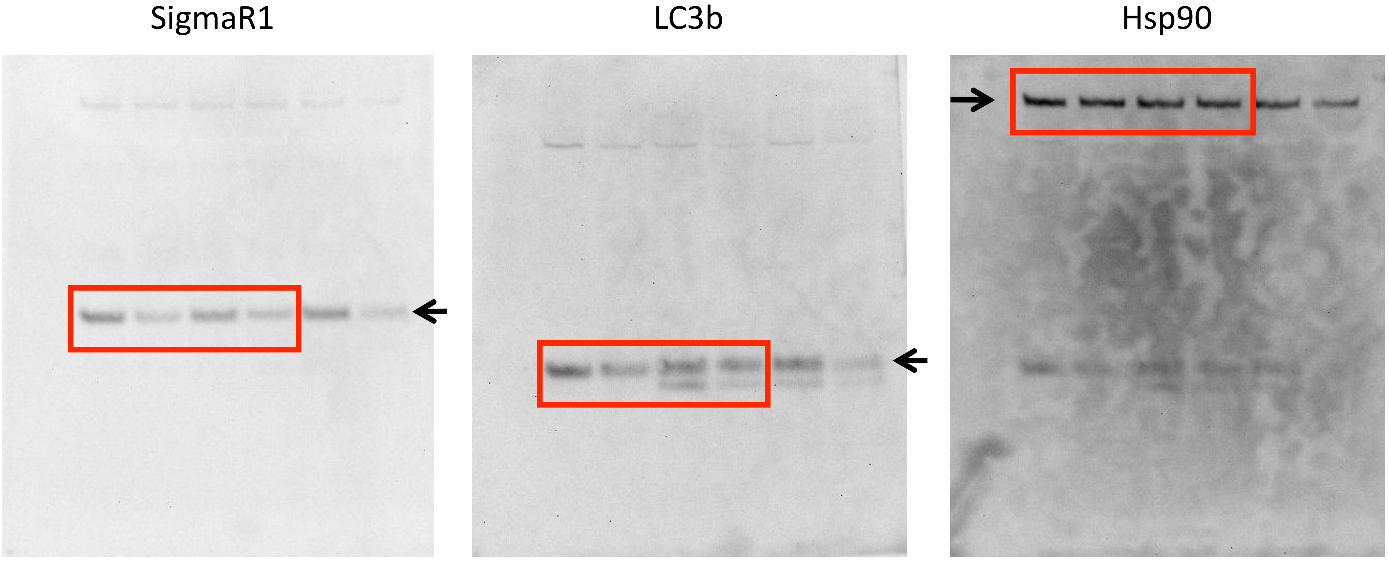

Figure S1A

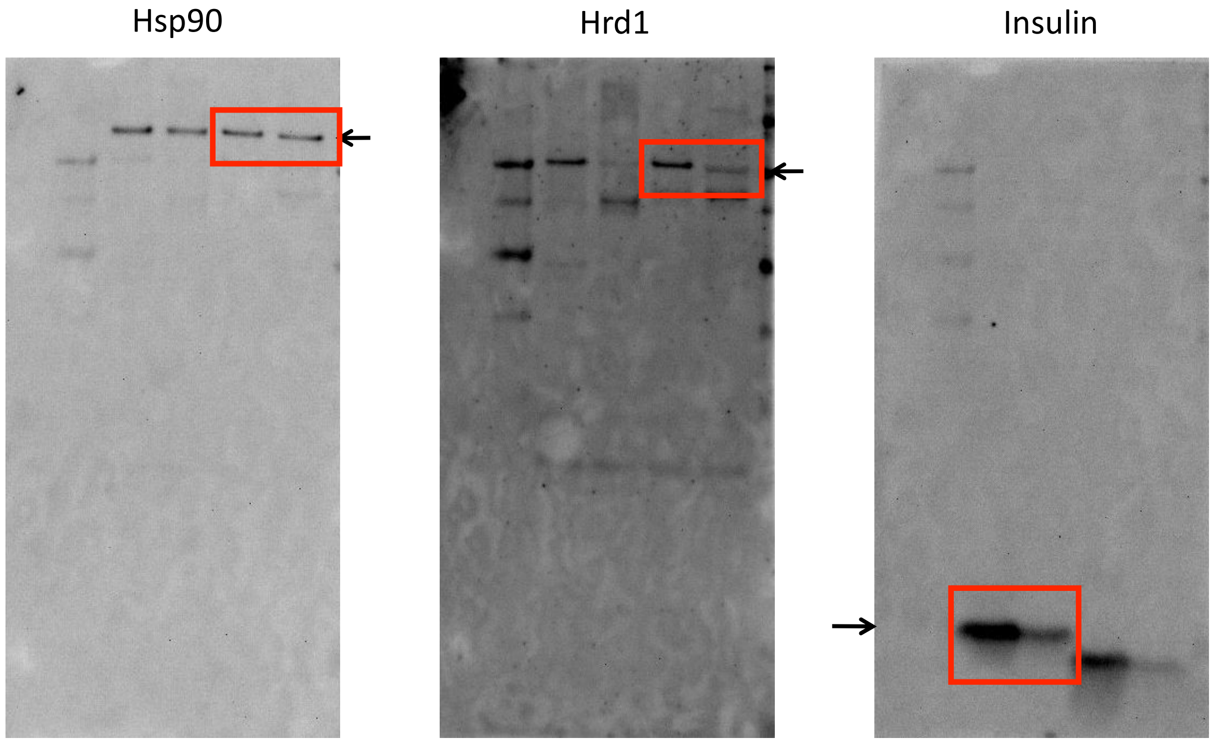

Figure S1C

Left

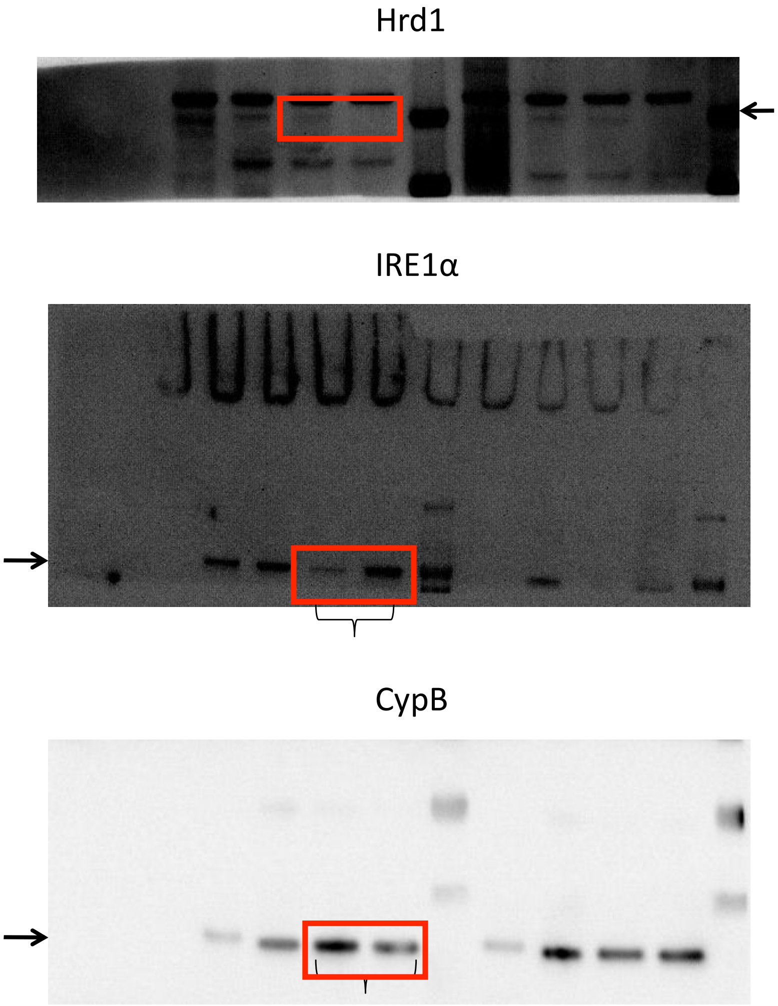

Figure S1C

Right

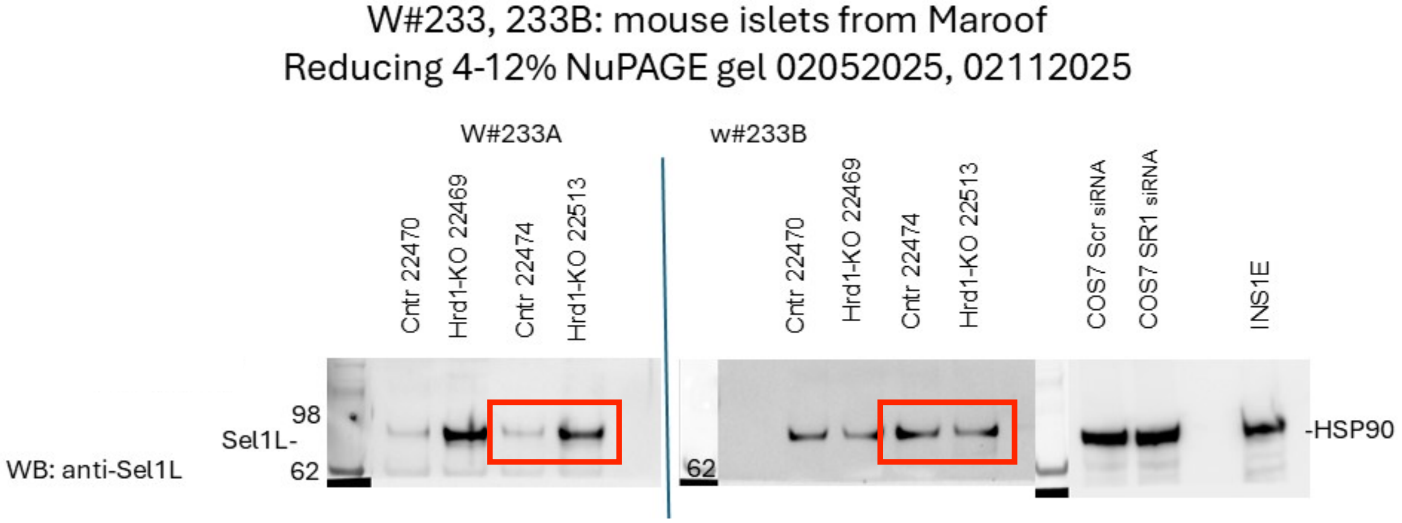

Figure S6

A

Pulse-chase analysis

mouse	Control			β-*HRD1*-KO				Control			β-*HRD1*-KO		
Cells (C) or media (M):	C	C	M	C	C	M		C	C	M	C	C	M
Chase time (h)	0	2	2	0	2	2		0	2	2	0	2	2
NR or R gel	NR	NR	NR	NR	NR	NR		R	R	R	R	R	R

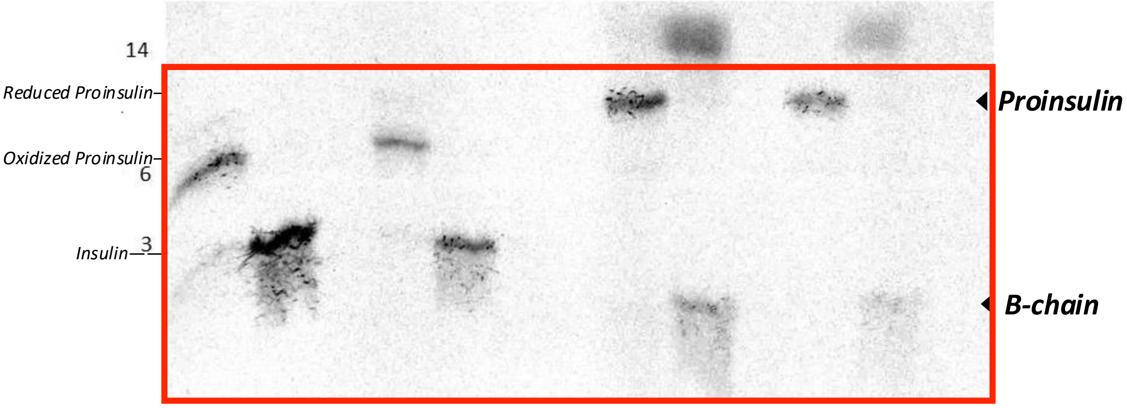

Figure S6B

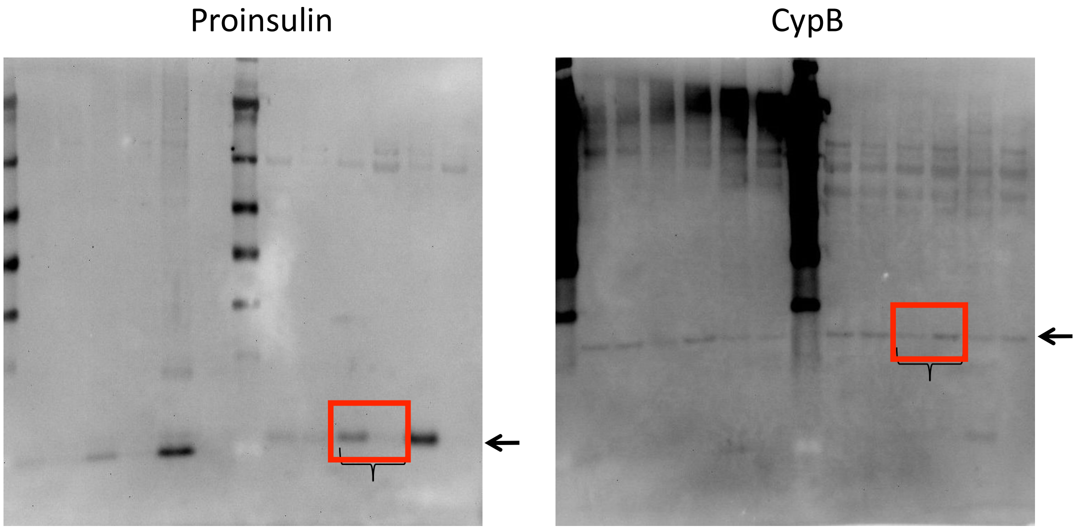

Figure S7A

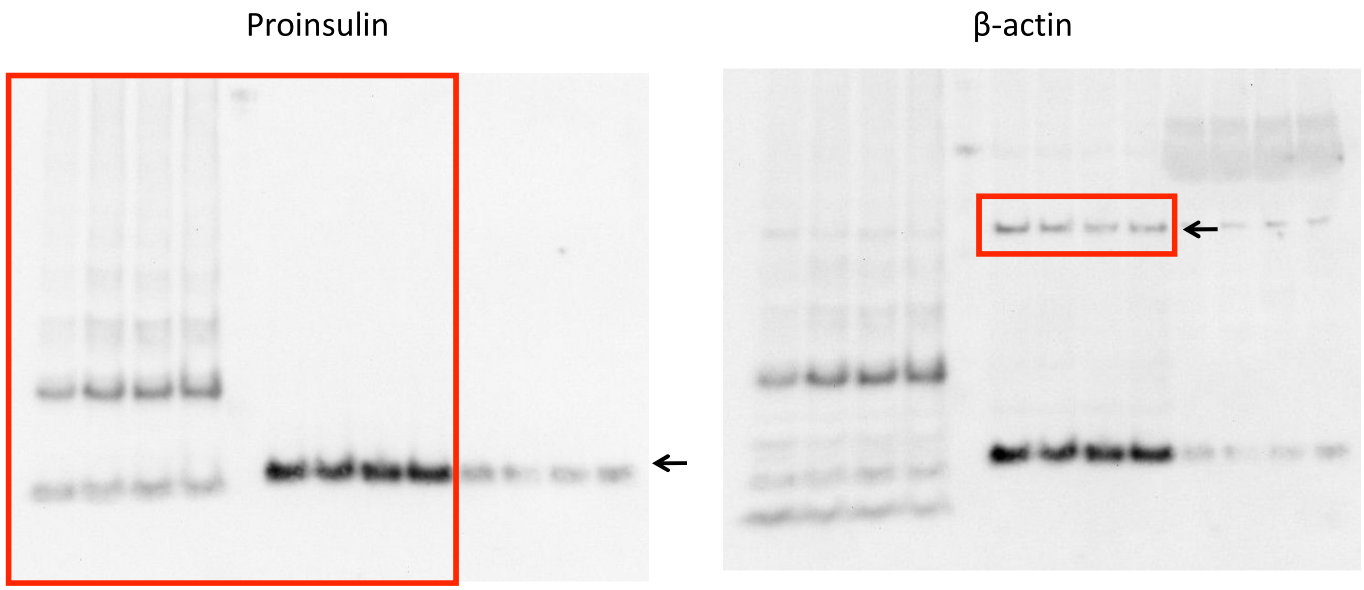

Figure S7B

Proinsulin

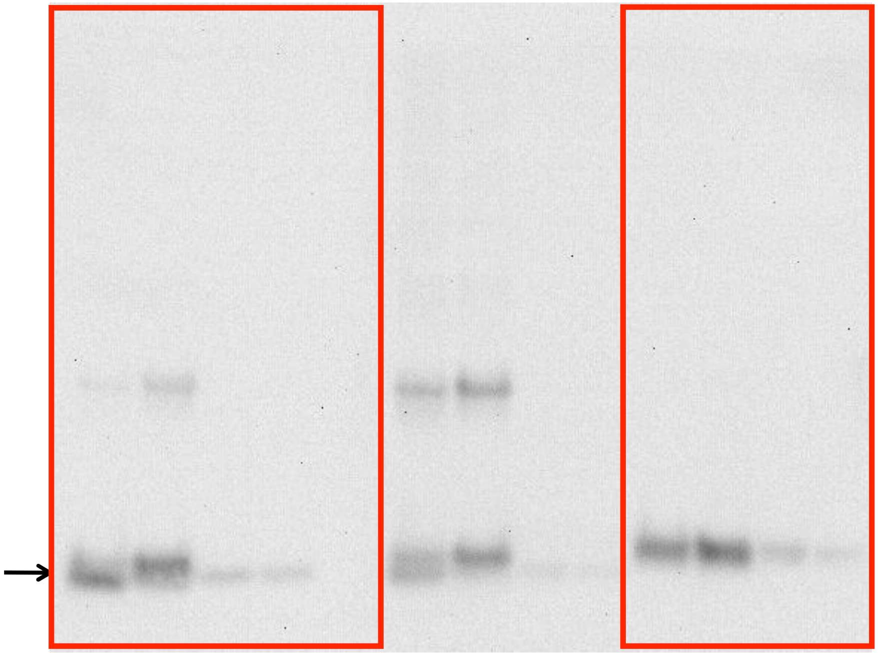

Figure S7C

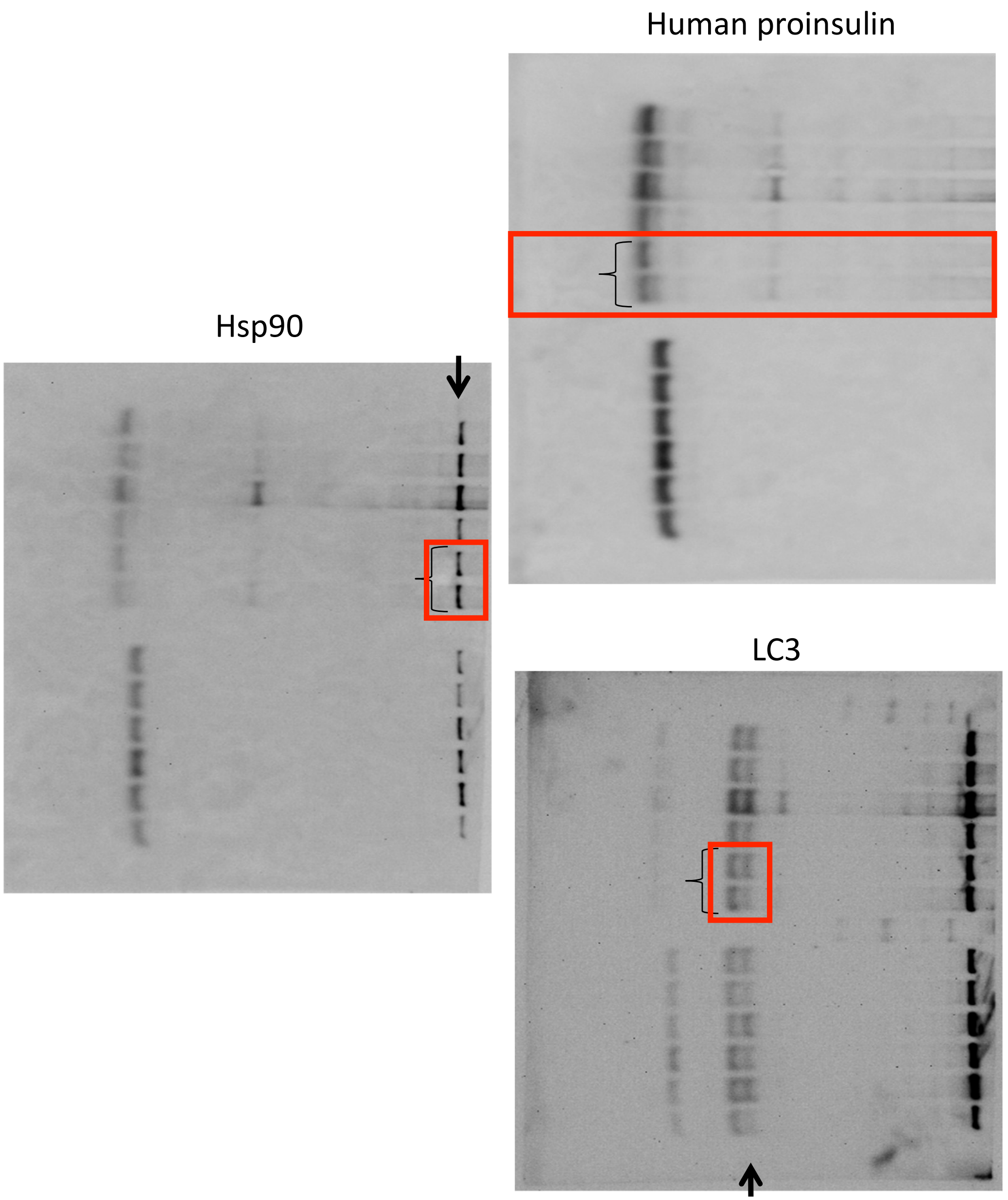

Figure S8A

Reduced Proinsulin

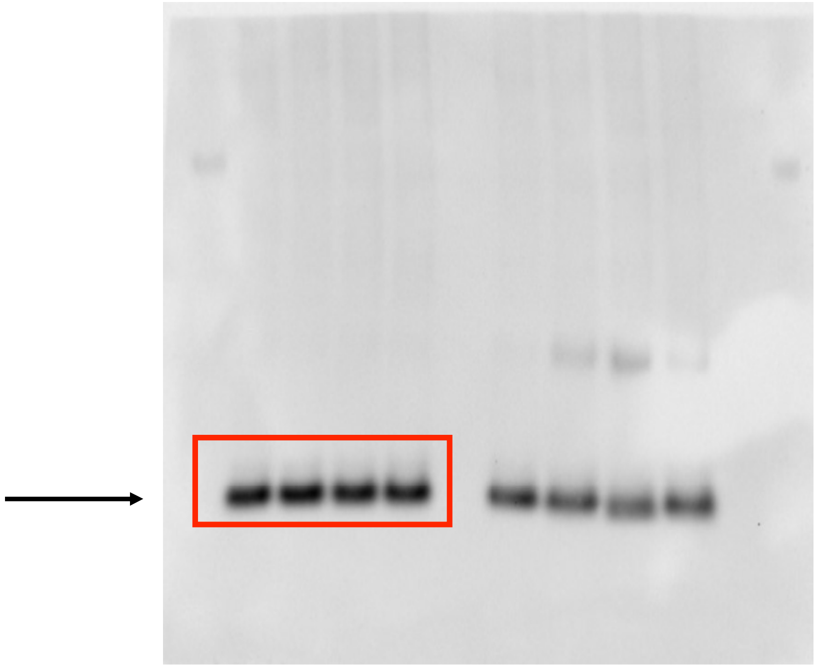

Figure S8A

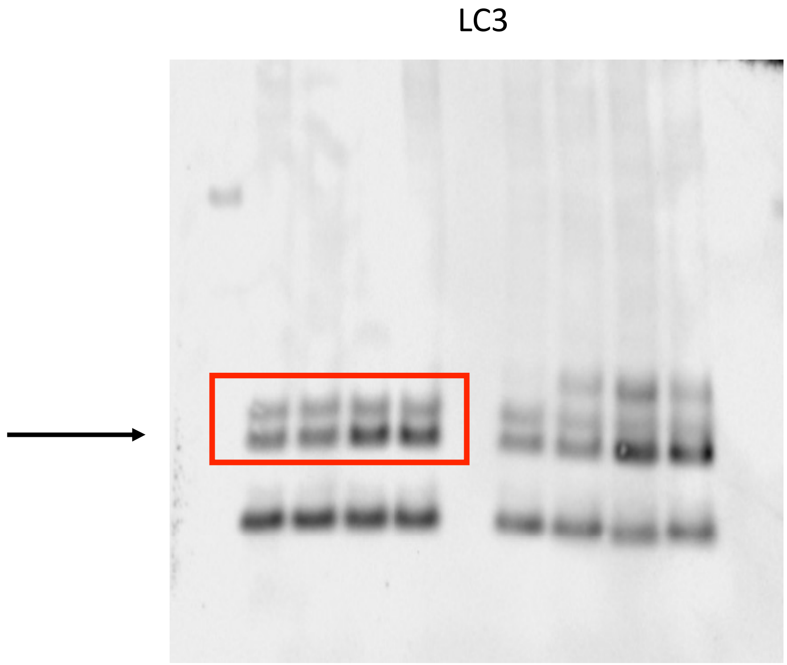

Figure S8A

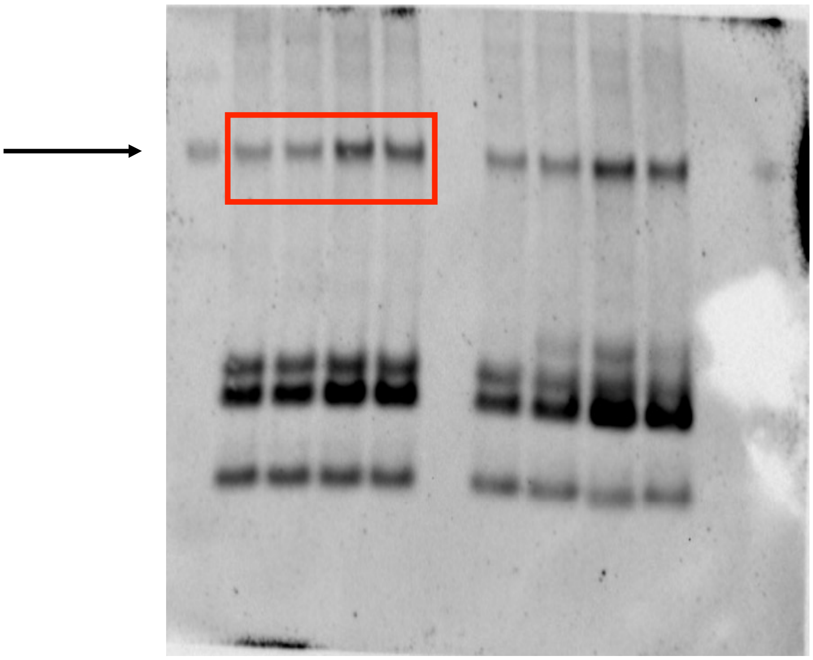

Figure S8A

Vinculin

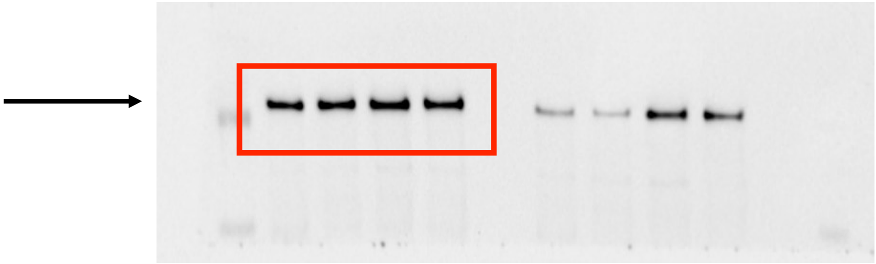

Figure S8B

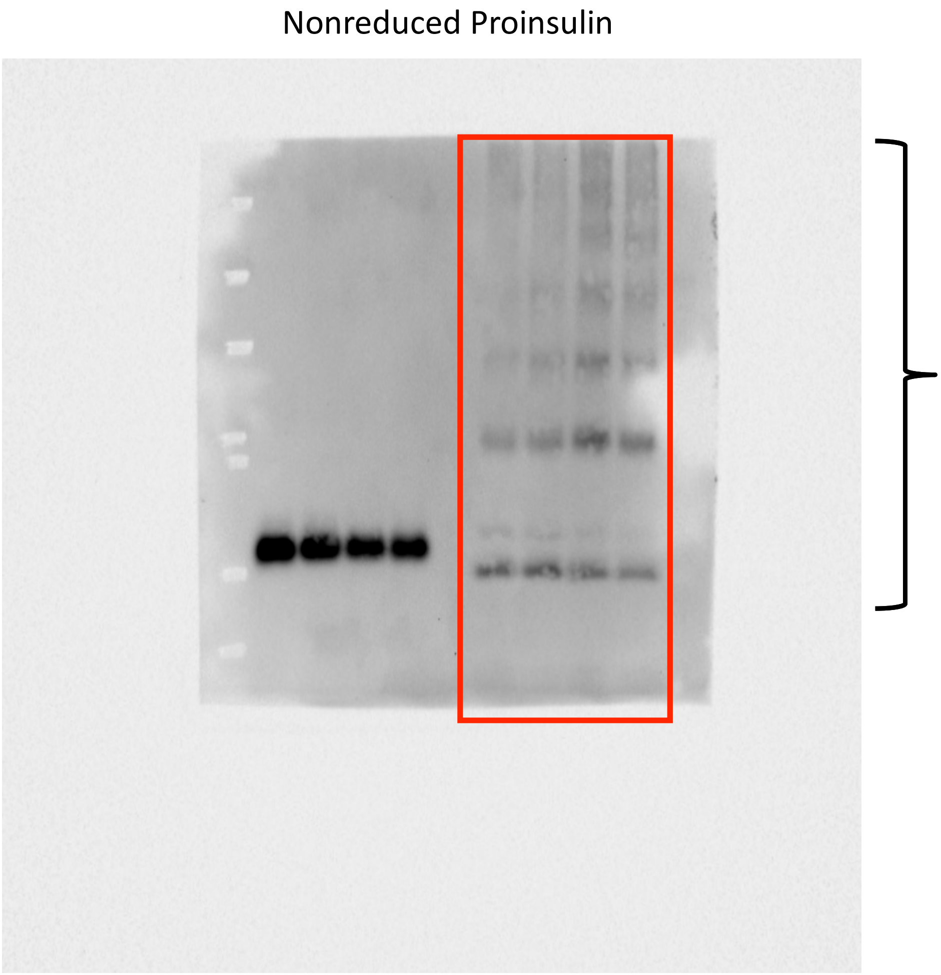

Figure S9

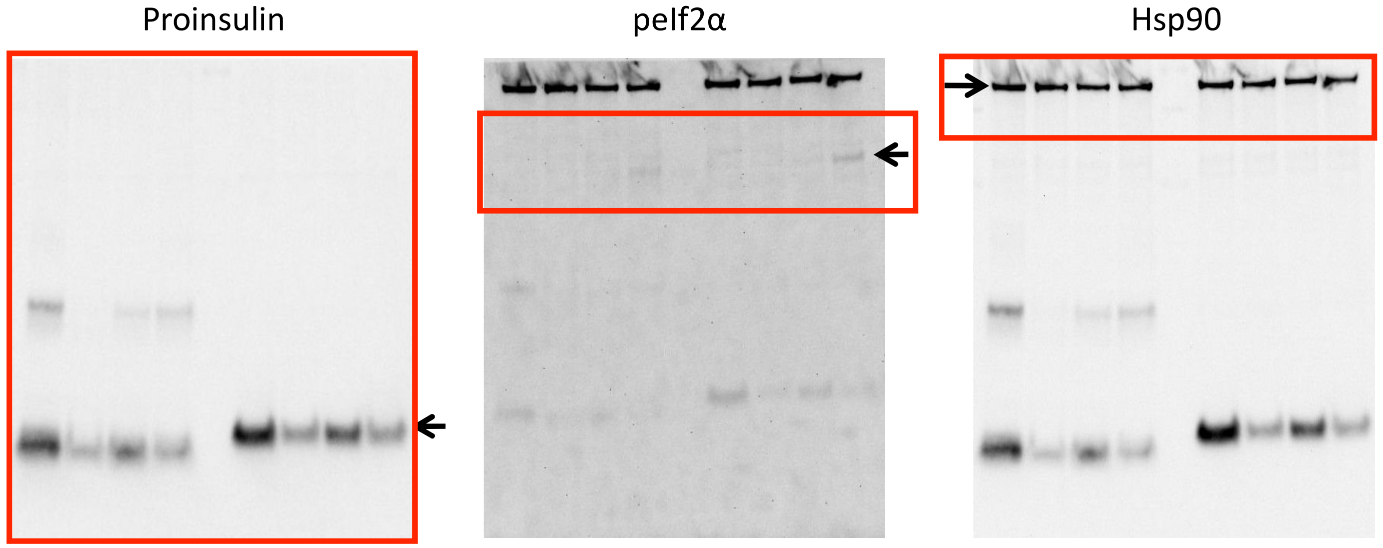

Figure S10A

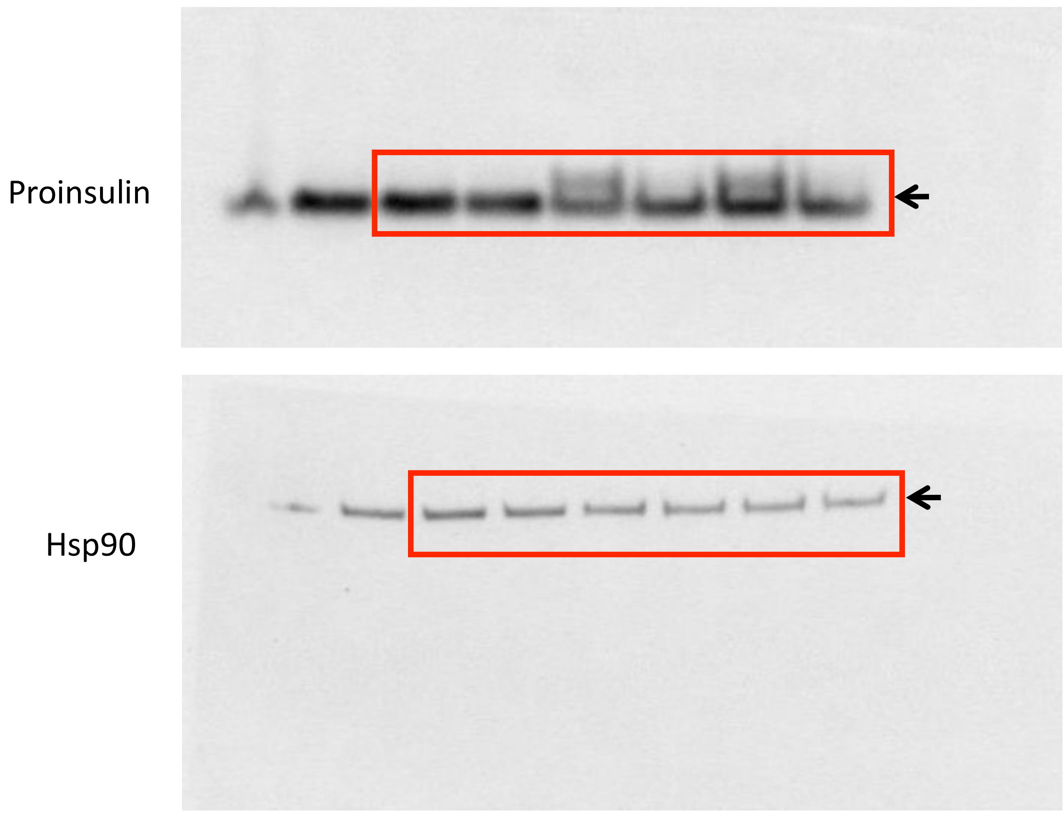

Figure S10B

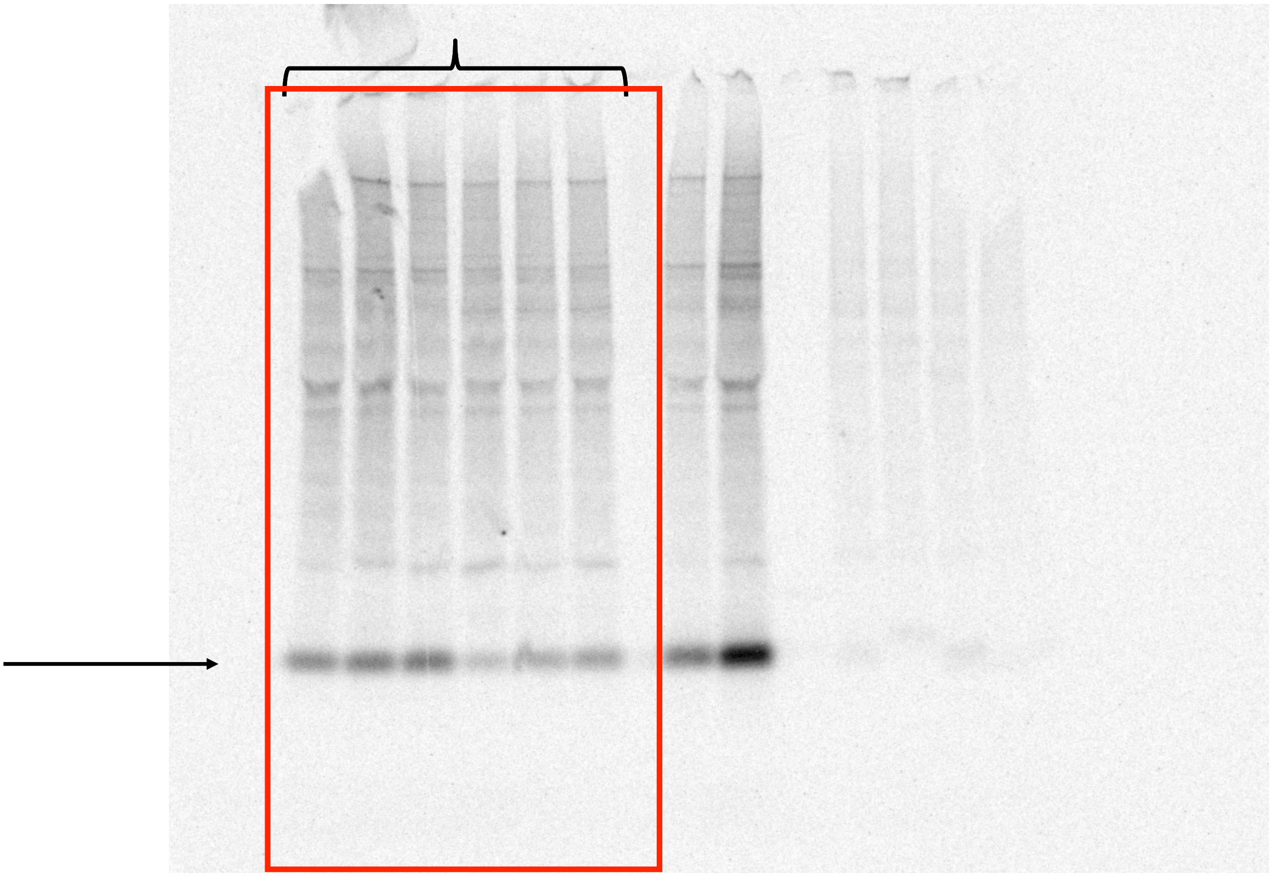

Figure S12A

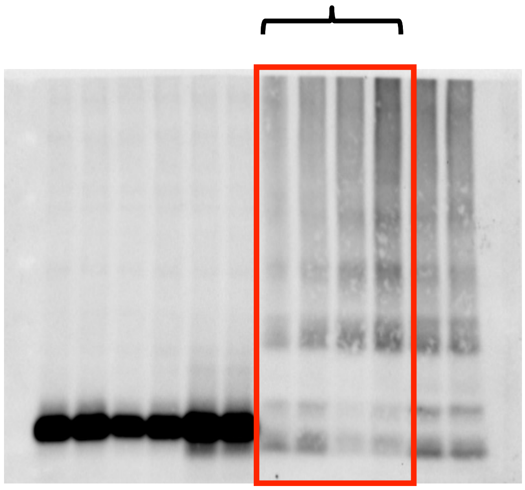

Figure S12A

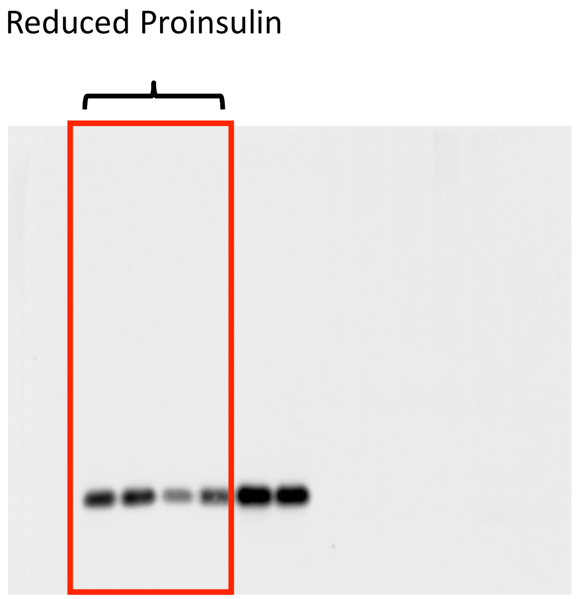

Figure S12A

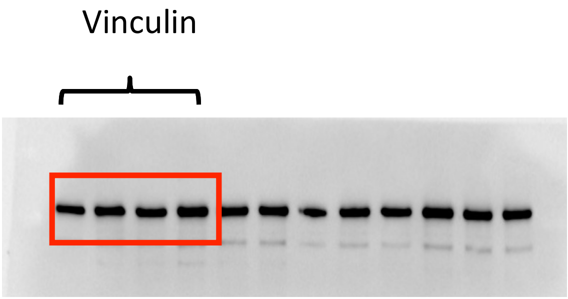

Figure S12D

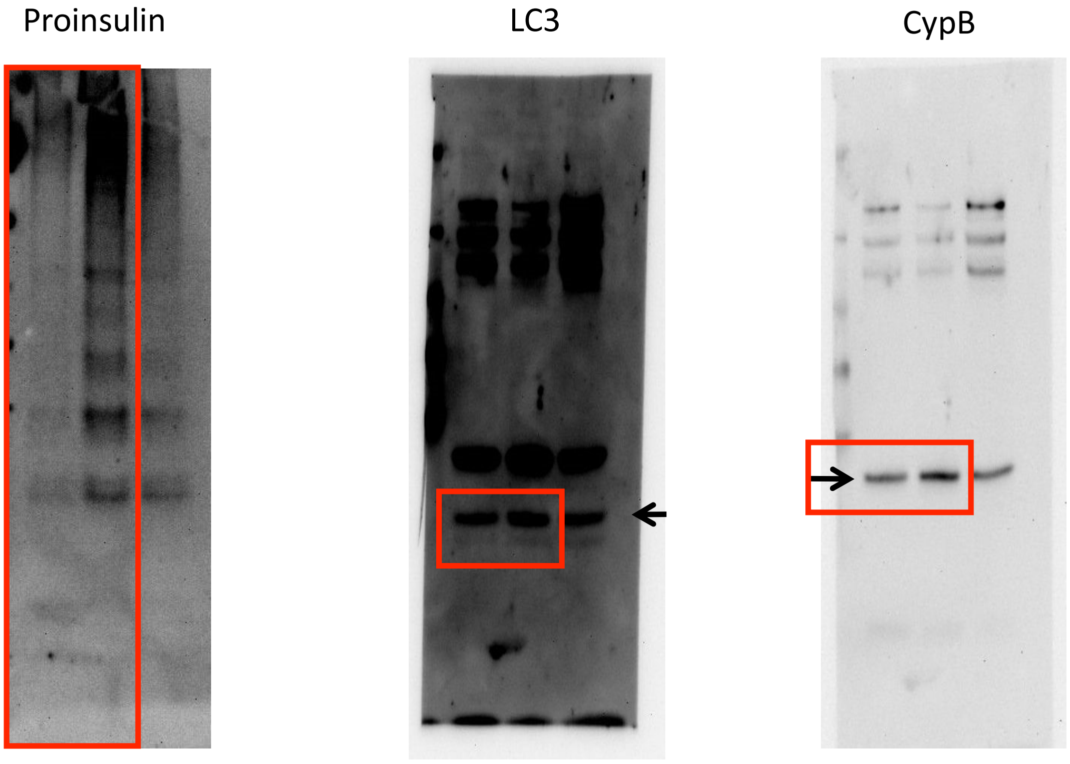

Figure S12E

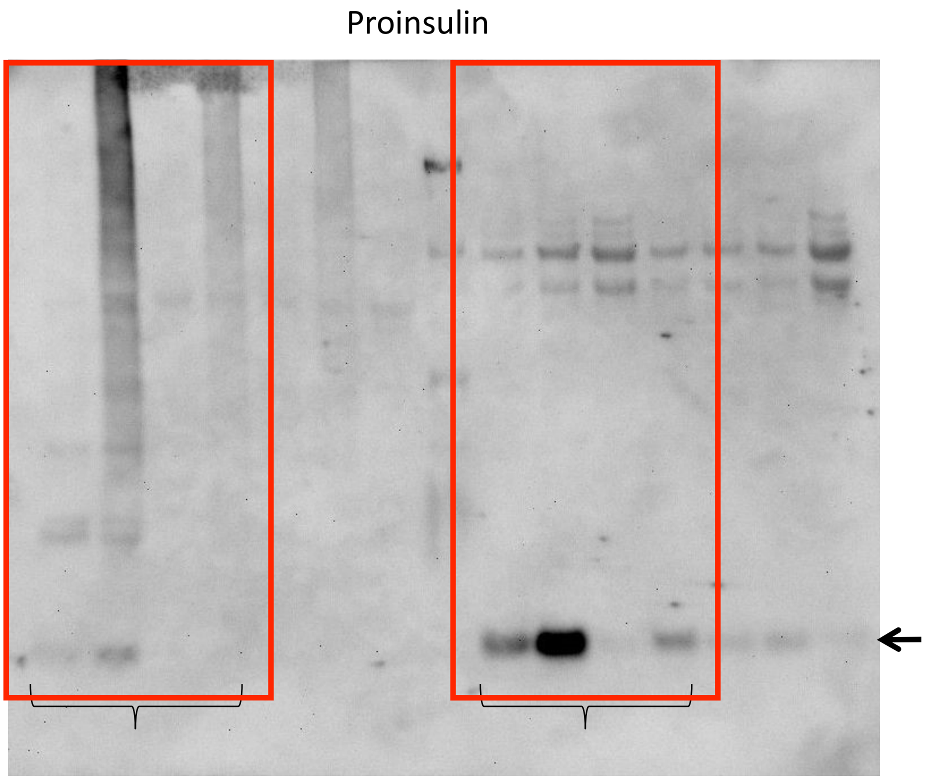

Figure S12E

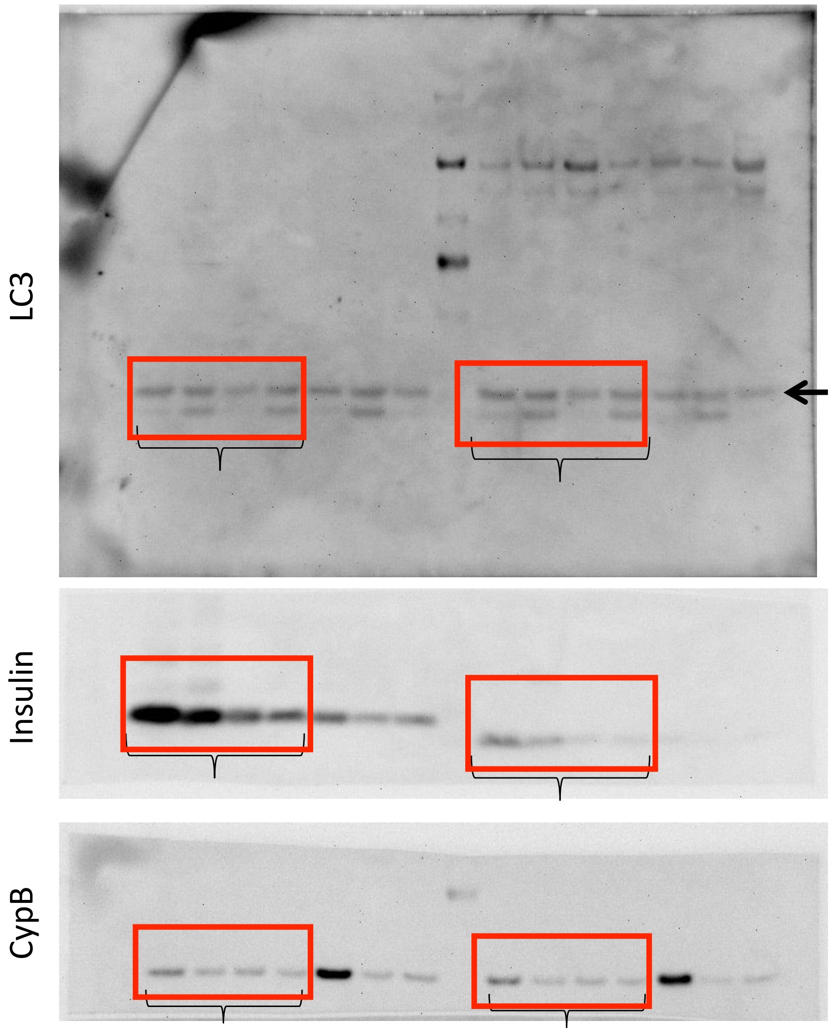

Supplement: Unedited blot and gel images [file jci-136-187341-s013.pdf]
